# Supplementary material for: Hearing of malaria mosquitoes is modulated by a beta-adrenergic-like octopamine receptor which serves as insecticide target
Source: Nat Commun. 2023 Jul 19;14:4338. doi: 10.1038/s41467-023-40029-y (PMC10356864; doi:10.1038/s41467-023-40029-y)
Supplement: Supplementary file 1 — Supplementary Information [file 41467_2023_40029_MOESM1_ESM.pdf]

Supplementary Information

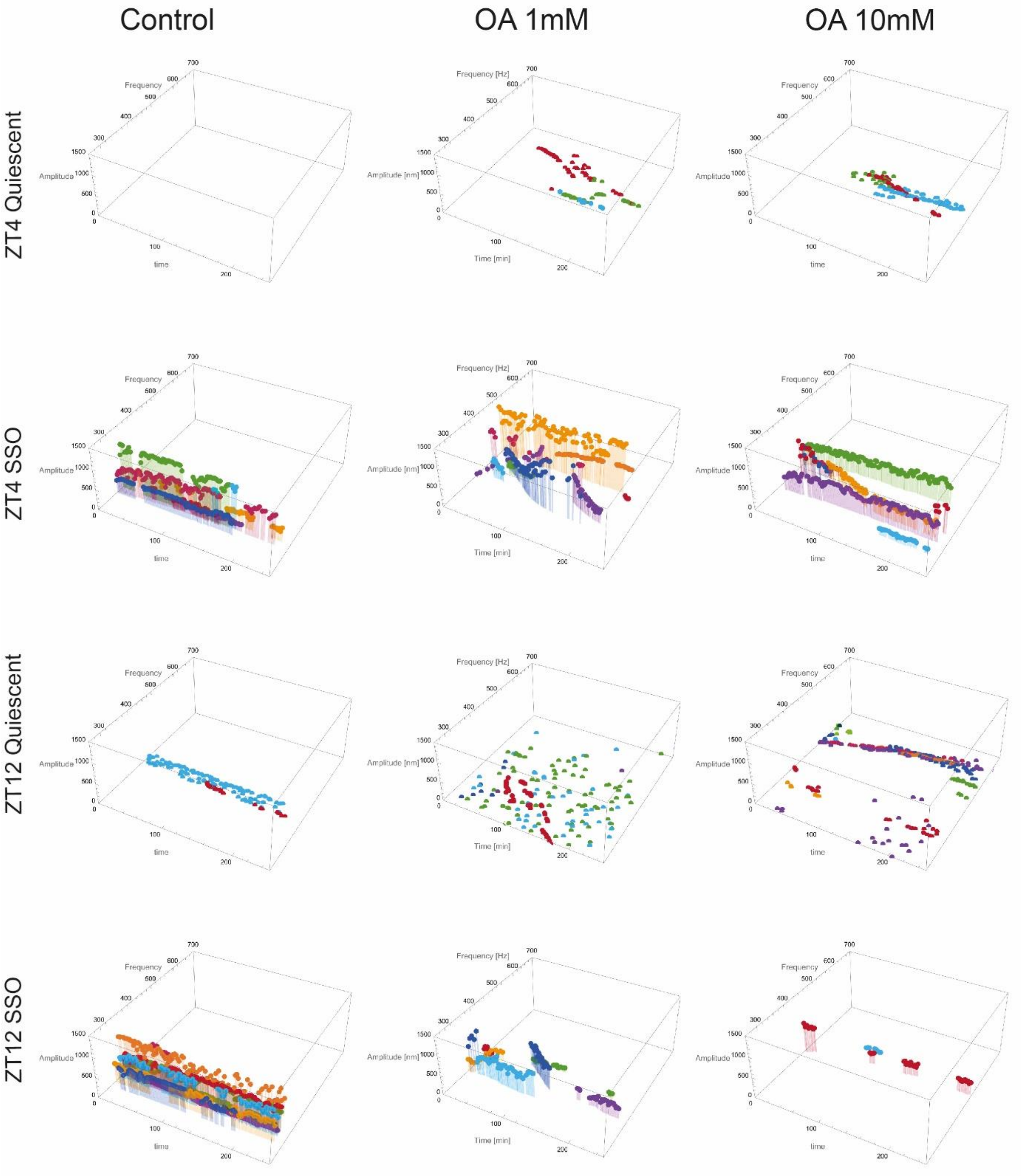

Supplementary Figure 1: Free fluctuation parameters for individual mosquitoes along the experimental recording. Frequency and amplitude of individual mosquito free fluctuations plotted against time for different conditions. Under control conditions, SSOing individual mosquitoes tend to maintain constant frequency and amplitude values. Overall, injecting octopamine caused remarkable shifts in free fluctuation values and a transition from SSO to quiescent mechanical states.

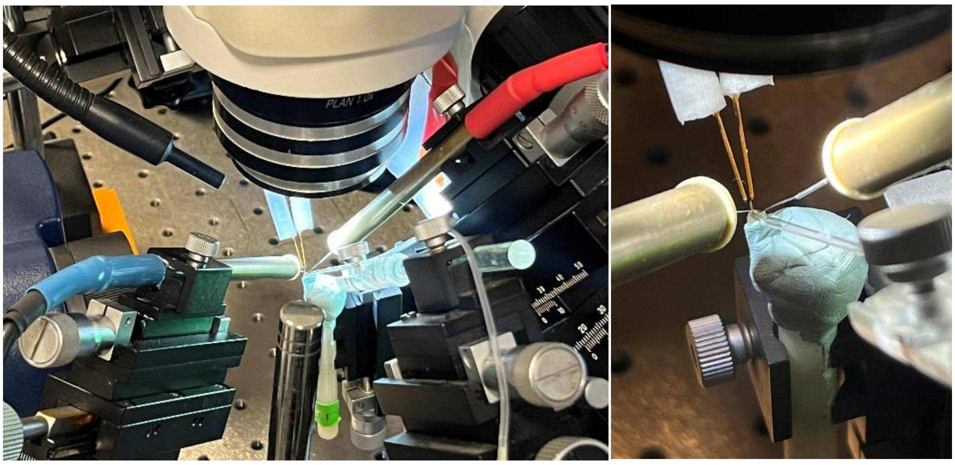

Supplementary Figure 2: Experimental setup for mosquito auditory tests. The mosquito was glued to a Teflon rod and placed in a micromanipulator on a vibration isolation table. The laser Doppler vibrometer was focused to the tip of the flagellum. A sharpened micro-capillary was inserted into the thorax to inject the different compounds. The micro-capillary was connected to a p20 pipette through a thin plastic pipe. A reference and recording electrode were inserted for electrophysiological measurements that are not reported in this manuscript.

Supplementary table 1: Summary of biophysical parameters extracted from antennal free fluctuations of SSO flagella in wildtype male mosquitoes upon exposure to octopamine

condition	State	parameter	mean	sd	median	mad
ZT4_G3_Ringer2	SSO	Frequency (Hz)	350.0115	10.84086	351.1111	6.589333
ZT4_G3_Ringer2	SSO	Amplitude (Hz)	519.4915	298.8118	409.0495	341.4709
ZT4_G3_OA1	SSO	Frequency (Hz)	491.5491	70.91821	517.7778	92.25067
ZT4_G3_OA1	SSO	Amplitude (Hz)	623.5885	430.3635	558.8202	577.1739
ZT4_G3_OA10	SSO	Frequency (Hz)	412.5452	57.22712	397.7778	52.71467
ZT4_G3_OA10	SSO	Amplitude (Hz)	414.074	129.6578	420.367	114.859
ZT12_G3_Ringer2	SSO	Frequency (Hz)	351.6738	23.56209	355.5556	9.884
ZT12_G3_Ringer2	SSO	Amplitude (Hz)	450.0125	235.6555	483.2234	176.4449
ZT12_G3_OA1	SSO	Frequency (Hz)	407.3978	41.94953	397.7778	52.71467
ZT12_G3_OA1	SSO	Amplitude (Hz)	386.1355	195.8682	390.6343	223.5977
ZT12_G3_OA10	SSO	Frequency (Hz)	510.2116	25.81783	508.8889	6.589333
ZT12_G3_OA10	SSO	Amplitude (Hz)	296.1106	201.9494	279.7756	68.33828

Supplementary table 2: Two-sided wilcoxon signed-rank test on free fluctuation analysis. The Holms procedure was used to correct for multiple comparisons.

parameter	group1	group2	p	p.adj	p.format	p.signif	method
Frequency	ZT4|Ringer2	ZT4|OA1	6.03E-106	8.40E-105	<2e-16	****	Wilcoxon
Frequency	ZT4|Ringer2	ZT4|OA10	1.63E-77	2.00E-76	<2e-16	****	Wilcoxon
Frequency	ZT4|Ringer2	ZT12|Ringer2	0.004813	0.014	0.0048	**	Wilcoxon
Frequency	ZT4|Ringer2	ZT12|OA1	1.52E-63	1.50E-62	<2e-16	****	Wilcoxon
Frequency	ZT4|Ringer2	ZT12|OA10	1.37E-26	8.20E-26	<2e-16	****	Wilcoxon
Frequency	ZT4|OA1	ZT4|OA10	8.05E-43	7.20E-42	<2e-16	****	Wilcoxon
Frequency	ZT4|OA1	ZT12|Ringer2	1.10E-128	1.60E-127	<2e-16	****	Wilcoxon
Frequency	ZT4|OA1	ZT12|OA1	1.20E-31	9.60E-31	<2e-16	****	Wilcoxon
Frequency	ZT4|OA1	ZT12|OA10	0.366508	0.67	0.3665	ns	Wilcoxon
Frequency	ZT4|OA10	ZT12|Ringer2	6.57E-104	8.50E-103	<2e-16	****	Wilcoxon
Frequency	ZT4|OA10	ZT12|OA1	0.335243	0.67	0.3352	ns	Wilcoxon
Frequency	ZT4|OA10	ZT12|OA10	9.85E-22	4.90E-21	<2e-16	****	Wilcoxon
Frequency	ZT12|Ringer2	ZT12|OA1	8.85E-68	9.70E-67	<2e-16	****	Wilcoxon
Frequency	ZT12|Ringer2	ZT12|OA10	7.23E-28	5.10E-27	<2e-16	****	Wilcoxon
Frequency	ZT12|OA1	ZT12|OA10	3.27E-21	1.30E-20	<2e-16	****	Wilcoxon
Amplitude	ZT4|Ringer2	ZT4|OA1	0.004444	0.022	0.00444	**	Wilcoxon
Amplitude	ZT4|Ringer2	ZT4|OA10	0.014661	0.039	0.01466	*	Wilcoxon
Amplitude	ZT4|Ringer2	ZT12|Ringer2	0.006153	0.025	0.00615	**	Wilcoxon
Amplitude	ZT4|Ringer2	ZT12|OA1	1.56E-05	0.00012	1.60E-05	****	Wilcoxon
Amplitude	ZT4|Ringer2	ZT12|OA10	1.27E-06	1.70E-05	1.30E-06	****	Wilcoxon
Amplitude	ZT4|OA1	ZT4|OA10	4.46E-08	6.70E-07	4.50E-08	****	Wilcoxon
Amplitude	ZT4|OA1	ZT12|Ringer2	2.85E-06	3.10E-05	2.80E-06	****	Wilcoxon
Amplitude	ZT4|OA1	ZT12|OA1	1.75E-06	2.10E-05	1.80E-06	****	Wilcoxon
Amplitude	ZT4|OA1	ZT12|OA10	6.33E-06	5.70E-05	6.30E-06	****	Wilcoxon
Amplitude	ZT4|OA10	ZT12|Ringer2	4.54E-06	4.50E-05	4.50E-06	****	Wilcoxon
Amplitude	ZT4|OA10	ZT12|OA1	0.136322	0.14	0.13632	ns	Wilcoxon

Amplitude	ZT4|OA10	ZT12|OA10	7.63E-07	1.10E-05	7.60E-07	****	Wilcoxon
Amplitude	ZT12|Ringer2	ZT12|OA1	0.002111	0.013	0.00211	**	Wilcoxon
Amplitude	ZT12|Ringer2	ZT12|OA10	0.000137	0.00096	0.00014	***	Wilcoxon
Amplitude	ZT12|OA1	ZT12|OA10	0.012948	0.039	0.01295	*	Wilcoxon

Supplementary table 3: Summary of biophysical parameters extracted from frequency-modulated sweep responses in wildtype male mosquitoes upon exposure to octopamine.

condition	State	parameters	mean	sd	median	mad
ZT4_G3_baseline	SSO	F0m	20.35273302	4.738395064	20.60716448	3.66299089
ZT4_G3_baseline	SSO	F0	359.1400548	30.1278469	354.9507098	31.96330078
ZT4_G3_baseline	SSO	peak	370.9728313	31.48362053	363.0579951	40.47507692
ZT4_G3_baseline	SSO	Q-factor	2.902706423	0.35218103	3.00520941	0.377750951
ZT4_G3_Ringer2	SSO	F0m	27.77139247	12.05081123	23.31791831	10.64343406
ZT4_G3_Ringer2	SSO	F0	365.6155117	45.40778588	343.1775424	42.24835089
ZT4_G3_Ringer2	SSO	peak	378.7454322	48.19835919	362.7802875	60.30172098
ZT4_G3_Ringer2	SSO	Q-factor	2.845145077	0.469116082	2.915655342	0.705523845
ZT4_G3_OA1	SSO	F0m	12.03601571	13.95091835	13.59297282	5.699110207
ZT4_G3_OA1	SSO	F0	515.086929	79.4073774	558.1754534	61.85895663
ZT4_G3_OA1	SSO	peak	594.1026338	584.6472938	563.0397569	78.75761598
ZT4_G3_OA1	SSO	Q-factor	4.251782294	1.674455097	4.108642686	1.947390688
ZT4_G3_OA1	Quiesc	F0m	-263.373394	1109.224111	10.04957154	6.914272074
ZT4_G3_OA1	Quiesc	F0	581.5118834	338.9819724	434.192478	43.42625597
ZT4_G3_OA1	Quiesc	peak	1023.012506	1618.843766	462.0237468	56.9774492
ZT4_G3_OA1	Quiesc	Q-factor	2.14792481	0.955847395	2.112563048	0.891434153
ZT4_G3_OA10	SSO	F0m	28.42117713	22.80354027	38.73630306	12.90141542
ZT4_G3_OA10	SSO	F0	451.2263967	139.6911688	447.8948417	111.9512385
ZT4_G3_OA10	SSO	peak	465.0128913	143.026154	460.0475045	119.6940598
ZT4_G3_OA10	SSO	Q-factor	3.06261927	0.755502696	2.976706776	0.225781695
ZT4_G3_OA10	Quiesc	F0m	108.5402517	557.6970959	19.70692441	17.01261236
ZT4_G3_OA10	Quiesc	F0	58	33.34166563	58	42.9954
ZT4_G3_OA10	Quiesc	peak	775.3942069	521.6927658	661.4783771	57.22864645

ZT4_G3_OA10	Quiesc	Q-factor	3.322728023	4.084738445	2.041192113	0.94011857
ZT12_G3_baseline	SSO	F0m	23.51784859	6.326110419	22.22667379	7.621279068
ZT12_G3_baseline	SSO	F0	358.995452	31.00066089	344.4432815	20.21407298
ZT12_G3_baseline	SSO	peak	374.8011826	37.59590604	356.361065	26.66236302
ZT12_G3_baseline	SSO	Q-factor	2.842306437	0.622395526	2.941332579	0.587244165
ZT12_G3_Ringer2	SSO	F0m	26.48755907	10.41536778	21.05205477	2.296166676
ZT12_G3_Ringer2	SSO	F0	364.1124947	32.85777706	349.9946001	16.86165395
ZT12_G3_Ringer2	SSO	peak	376.4643014	33.64256422	359.6971497	13.29781816
ZT12_G3_Ringer2	SSO	Q-factor	2.843248208	0.321678387	2.889792006	0.39536437
ZT12_G3_Ringer2	Quiesc	F0m	28.55763792	5.891399163	26.09950007	1.347586502
ZT12_G3_Ringer2	Quiesc	F0	362.4659264	57.17623454	335.2486033	3.224514216
ZT12_G3_Ringer2	Quiesc	peak	377.4271871	58.95408573	349.203811	3.112135767
ZT12_G3_Ringer2	Quiesc	Q-factor	2.536402775	NA	2.571113148	0.087909052
ZT12_G3_OA1	SSO	F0m	-11.24262818	356.0904924	20.44884551	4.165593337
ZT12_G3_OA1	SSO	F0	402.8884611	139.1632794	382.3274199	46.89736817
ZT12_G3_OA1	SSO	peak	461.7241749	547.9159479	417.2433041	66.91408303
ZT12_G3_OA1	SSO	Q-factor	2.556991848	0.624290284	2.791319055	0.472191449
ZT12_G3_OA1	Quiesc	F0m	-16.25789799	354.9364274	14.31720488	18.76890668
ZT12_G3_OA1	Quiesc	F0	552.244119	676.2422417	544.5223919	108.0511881
ZT12_G3_OA1	Quiesc	peak	0.400546949	253.8425574	553.2625261	94.26433771
ZT12_G3_OA1	Quiesc	Q-factor	5.213208498	6.134360618	2.279820899	1.052797248
ZT12_G3_OA10	SSO	F0m	26.34493001	16.75960454	18.79455516	0.734745888
ZT12_G3_OA10	SSO	F0	549.1575731	84.91681174	502.9245365	15.71099862
ZT12_G3_OA10	SSO	peak	574.9574307	102.1111928	521.0065656	17.10435797

ZT12_G3_OA10	SSO	Q-factor	4.259433766	9.404622673	2.697153745	0.092956817
ZT12_G3_OA10	Quiesc	F0m	22.8824558	806.090336	-0.222509568	5.7408845
ZT12_G3_OA10	Quiesc	F0	542.6326169	707.8395543	393.6803836	172.1192847
ZT12_G3_OA10	Quiesc	peak	643.1923444	608.3057539	554.8580366	158.7481815
ZT12_G3_OA10	Quiesc	Q-factor	4.035857682	7.715059691	1.109098167	0.459045342

Supplementary table 4: Two-sided wilcoxon signed-rank test on frequency-modulated sweep analysis in males (pairwise comparisons). The Holms procedure was used to correct for multiple comparisons.

Parameter	group1	group2	p	p.adj	p.format	p.signif	method
F0m	ZT4|baseline|SSO	ZT4|control|SSO	2.45E-19	1.10E-17	< 2e-16	****	Wilcoxon
F0m	ZT4|baseline|SSO	ZT4|1mM OA|SSO	2.20E-41	1.40E-39	< 2e-16	****	Wilcoxon
F0m	ZT4|baseline|SSO	ZT4|1mM OA|Quiescent	4.39E-25	2.20E-23	< 2e-16	****	Wilcoxon
F0m	ZT4|baseline|SSO	ZT4|10mM OA|SSO	1.04E-19	4.90E-18	< 2e-16	****	Wilcoxon
F0m	ZT4|baseline|SSO	ZT4|10mM OA|Quiescent	0.34945	1	0.34945	ns	Wilcoxon
F0m	ZT4|baseline|SSO	ZT12|baseline|SSO	2.03E-06	5.30E-05	2.00E-06	****	Wilcoxon
F0m	ZT4|baseline|SSO	ZT12|control|SSO	6.64E-20	3.20E-18	< 2e-16	****	Wilcoxon
F0m	ZT4|baseline|SSO	ZT12|control|Quiescent	1.31E-30	7.50E-29	< 2e-16	****	Wilcoxon
F0m	ZT4|baseline|SSO	ZT12|1mM OA|SSO	0.146935	1	0.14694	ns	Wilcoxon
F0m	ZT4|baseline|SSO	ZT12|1mM OA|Quiescent	1.66E-36	1.00E-34	< 2e-16	****	Wilcoxon
F0m	ZT4|baseline|SSO	ZT12|10mM OA|SSO	0.890448	1	0.89045	ns	Wilcoxon
F0m	ZT4|baseline|SSO	ZT12|10mM OA|Quiescent	1.70E-63	1.30E-61	< 2e-16	****	Wilcoxon
F0m	ZT4|control|SSO	ZT4|1mM OA|SSO	3.15E-53	2.20E-51	< 2e-16	****	Wilcoxon
F0m	ZT4|control|SSO	ZT4|1mM OA|Quiescent	6.27E-33	3.70E-31	< 2e-16	****	Wilcoxon
F0m	ZT4|control|SSO	ZT4|10mM OA|SSO	1.57E-07	4.70E-06	1.60E-07	****	Wilcoxon

F0m	ZT4|control|SSO	ZT4|10mM OA|Quiescent	7.06E-07	2.00E-05	7.10E-07	****	Wilcoxon
F0m	ZT4|control|SSO	ZT12|baseline|SSO	0.08659	0.95	0.08659	ns	Wilcoxon
F0m	ZT4|control|SSO	ZT12|control|SSO	0.02855	0.4	0.02855	*	Wilcoxon
F0m	ZT4|control|SSO	ZT12|control|Quiescent	0.000685	0.013	0.00068	***	Wilcoxon
F0m	ZT4|control|SSO	ZT12|1mM OA|SSO	5.72E-05	0.0012	5.70E-05	****	Wilcoxon
F0m	ZT4|control|SSO	ZT12|1mM OA|Quiescent	3.06E-39	1.90E-37	< 2e-16	****	Wilcoxon
F0m	ZT4|control|SSO	ZT12|10mM OA|SSO	0.059736	0.72	0.05974	ns	Wilcoxon
F0m	ZT4|control|SSO	ZT12|10mM OA|Quiescent	3.32E-63	2.50E-61	< 2e-16	****	Wilcoxon
F0m	ZT4|1mM OA|SSO	ZT4|1mM OA|Quiescent	4.05E-05	0.00089	4.10E-05	****	Wilcoxon
F0m	ZT4|1mM OA|SSO	ZT4|10mM OA|SSO	9.09E-50	6.10E-48	< 2e-16	****	Wilcoxon
F0m	ZT4|1mM OA|SSO	ZT4|10mM OA|Quiescent	0.000169	0.0034	0.00017	***	Wilcoxon
F0m	ZT4|1mM OA|SSO	ZT12|baseline|SSO	9.01E-51	6.10E-49	< 2e-16	****	Wilcoxon
F0m	ZT4|1mM OA|SSO	ZT12|control|SSO	1.04E-84	8.00E-83	< 2e-16	****	Wilcoxon
F0m	ZT4|1mM OA|SSO	ZT12|control|Quiescent	1.65E-49	1.10E-47	< 2e-16	****	Wilcoxon
F0m	ZT4|1mM OA|SSO	ZT12|1mM OA|SSO	3.85E-30	2.20E-28	< 2e-16	****	Wilcoxon
F0m	ZT4|1mM OA|SSO	ZT12|1mM OA|Quiescent	0.018034	0.29	0.01803	*	Wilcoxon
F0m	ZT4|1mM OA|SSO	ZT12|10mM OA|SSO	7.70E-10	2.80E-08	7.70E-10	****	Wilcoxon
F0m	ZT4|1mM OA|SSO	ZT12|10mM OA|Quiescent	2.02E-22	9.90E-21	< 2e-16	****	Wilcoxon
F0m	ZT4|1mM OA|Quiescent	ZT4|10mM OA|SSO	1.17E-27	6.30E-26	< 2e-16	****	Wilcoxon
F0m	ZT4|1mM OA|Quiescent	ZT4|10mM OA|Quiescent	1.05E-05	0.00024	1.10E-05	****	Wilcoxon
F0m	ZT4|1mM OA|Quiescent	ZT12|baseline|SSO	2.94E-32	1.70E-30	< 2e-16	****	Wilcoxon
F0m	ZT4|1mM OA|Quiescent	ZT12|control|SSO	2.66E-35	1.60E-33	< 2e-16	****	Wilcoxon
F0m	ZT4|1mM OA|Quiescent	ZT12|control|Quiescent	1.40E-28	7.70E-27	< 2e-16	****	Wilcoxon
F0m	ZT4|1mM OA|Quiescent	ZT12|1mM OA|SSO	6.45E-23	3.20E-21	< 2e-16	****	Wilcoxon
F0m	ZT4|1mM OA|Quiescent	ZT12|1mM OA|Quiescent	0.007903	0.13	0.0079	**	Wilcoxon

F0m	ZT4|1mM OA|Quiescent	ZT12|10mM OA|SSO	2.16E-12	9.10E-11	2.20E-12	****	Wilcoxon
F0m	ZT4|1mM OA|Quiescent	ZT12|10mM OA|Quiescent	0.021742	0.33	0.02174	*	Wilcoxon
F0m	ZT4|10mM OA|SSO	ZT4|10mM OA|Quiescent	2.39E-10	9.10E-09	2.40E-10	****	Wilcoxon
F0m	ZT4|10mM OA|SSO	ZT12|baseline|SSO	1.15E-18	5.20E-17	< 2e-16	****	Wilcoxon
F0m	ZT4|10mM OA|SSO	ZT12|control|SSO	0.044202	0.57	0.0442	*	Wilcoxon
F0m	ZT4|10mM OA|SSO	ZT12|control|Quiescent	0.221555	1	0.22155	ns	Wilcoxon
F0m	ZT4|10mM OA|SSO	ZT12|1mM OA|SSO	4.25E-09	1.50E-07	4.30E-09	****	Wilcoxon
F0m	ZT4|10mM OA|SSO	ZT12|1mM OA|Quiescent	1.64E-55	1.20E-53	< 2e-16	****	Wilcoxon
F0m	ZT4|10mM OA|SSO	ZT12|10mM OA|SSO	0.137974	1	0.13797	ns	Wilcoxon
F0m	ZT4|10mM OA|SSO	ZT12|10mM OA|Quiescent	2.97E-52	2.10E-50	< 2e-16	****	Wilcoxon
F0m	ZT4|10mM OA|Quiescent	ZT12|baseline|SSO	0.002068	0.037	0.00207	**	Wilcoxon
F0m	ZT4|10mM OA|Quiescent	ZT12|control|SSO	7.93E-07	2.20E-05	7.90E-07	****	Wilcoxon
F0m	ZT4|10mM OA|Quiescent	ZT12|control|Quiescent	1.84E-10	7.20E-09	1.80E-10	****	Wilcoxon
F0m	ZT4|10mM OA|Quiescent	ZT12|1mM OA|SSO	0.174533	1	0.17453	ns	Wilcoxon
F0m	ZT4|10mM OA|Quiescent	ZT12|1mM OA|Quiescent	4.01E-06	1.00E-04	4.00E-06	****	Wilcoxon
F0m	ZT4|10mM OA|Quiescent	ZT12|10mM OA|SSO	0.288244	1	0.28824	ns	Wilcoxon
F0m	ZT4|10mM OA|Quiescent	ZT12|10mM OA|Quiescent	2.42E-10	9.10E-09	2.40E-10	****	Wilcoxon
F0m	ZT12|baseline|SSO	ZT12|control|SSO	1.51E-08	4.80E-07	1.50E-08	****	Wilcoxon
F0m	ZT12|baseline|SSO	ZT12|control|Quiescent	6.24E-11	2.50E-09	6.20E-11	****	Wilcoxon
F0m	ZT12|baseline|SSO	ZT12|1mM OA|SSO	0.387475	1	0.38747	ns	Wilcoxon
F0m	ZT12|baseline|SSO	ZT12|1mM OA|Quiescent	5.46E-51	3.80E-49	< 2e-16	****	Wilcoxon
F0m	ZT12|baseline|SSO	ZT12|10mM OA|SSO	0.934094	1	0.93409	ns	Wilcoxon
F0m	ZT12|baseline|SSO	ZT12|10mM OA|Quiescent	2.27E-54	1.60E-52	< 2e-16	****	Wilcoxon
F0m	ZT12|control|SSO	ZT12|control|Quiescent	2.14E-15	9.40E-14	2.10E-15	****	Wilcoxon
F0m	ZT12|control|SSO	ZT12|1mM OA|SSO	9.40E-09	3.10E-07	9.40E-09	****	Wilcoxon

F0m	ZT12|control|SSO	ZT12|1mM OA|Quiescent	1.81E-78	1.40E-76	< 2e-16	****	Wilcoxon
F0m	ZT12|control|SSO	ZT12|10mM OA|SSO	1.13E-06	3.10E-05	1.10E-06	****	Wilcoxon
F0m	ZT12|control|SSO	ZT12|10mM OA|Quiescent	2.37E-107	1.80E-105	< 2e-16	****	Wilcoxon
F0m	ZT12|control|Quiescent	ZT12|1mM OA|SSO	1.42E-11	5.80E-10	1.40E-11	****	Wilcoxon
F0m	ZT12|control|Quiescent	ZT12|1mM OA|Quiescent	1.35E-41	8.80E-40	< 2e-16	****	Wilcoxon
F0m	ZT12|control|Quiescent	ZT12|10mM OA|SSO	8.55E-06	0.00021	8.60E-06	****	Wilcoxon
F0m	ZT12|control|Quiescent	ZT12|10mM OA|Quiescent	2.42E-40	1.50E-38	< 2e-16	****	Wilcoxon
F0m	ZT12|1mM OA|SSO	ZT12|1mM OA|Quiescent	1.38E-25	7.30E-24	< 2e-16	****	Wilcoxon
F0m	ZT12|1mM OA|SSO	ZT12|10mM OA|SSO	0.30995	1	0.30995	ns	Wilcoxon
F0m	ZT12|1mM OA|SSO	ZT12|10mM OA|Quiescent	2.44E-25	1.30E-23	< 2e-16	****	Wilcoxon
F0m	ZT12|1mM OA|Quiescent	ZT12|10mM OA|SSO	6.77E-09	2.30E-07	6.80E-09	****	Wilcoxon
F0m	ZT12|1mM OA|Quiescent	ZT12|10mM OA|Quiescent	5.41E-13	2.30E-11	5.40E-13	****	Wilcoxon
F0m	ZT12|10mM OA|SSO	ZT12|10mM OA|Quiescent	2.80E-08	8.70E-07	2.80E-08	****	Wilcoxon
f0	ZT4|baseline|SSO	ZT4|control|SSO	0.638558	1	0.63856	ns	Wilcoxon
f0	ZT4|baseline|SSO	ZT4|1mM OA|SSO	6.96E-85	5.10E-83	< 2e-16	****	Wilcoxon
f0	ZT4|baseline|SSO	ZT4|1mM OA|Quiescent	2.49E-40	1.50E-38	< 2e-16	****	Wilcoxon
f0	ZT4|baseline|SSO	ZT4|10mM OA|SSO	2.59E-21	1.00E-19	< 2e-16	****	Wilcoxon
f0	ZT4|baseline|SSO	ZT4|10mM OA|Quiescent	2.93E-57	1.90E-55	< 2e-16	****	Wilcoxon
f0	ZT4|baseline|SSO	ZT12|baseline|SSO	0.097019	1	0.09702	ns	Wilcoxon
f0	ZT4|baseline|SSO	ZT12|control|SSO	1.10E-05	0.00023	1.10E-05	****	Wilcoxon
f0	ZT4|baseline|SSO	ZT12|control|Quiescent	0.019414	0.32	0.01941	*	Wilcoxon
f0	ZT4|baseline|SSO	ZT12|1mM OA|SSO	4.22E-19	1.40E-17	< 2e-16	****	Wilcoxon
f0	ZT4|baseline|SSO	ZT12|1mM OA|Quiescent	2.35E-97	1.80E-95	< 2e-16	****	Wilcoxon
f0	ZT4|baseline|SSO	ZT12|10mM OA|SSO	9.89E-23	4.40E-21	< 2e-16	****	Wilcoxon
f0	ZT4|baseline|SSO	ZT12|10mM OA|Quiescent	1.51E-103	1.10E-101	< 2e-16	****	Wilcoxon

<table>
<tr><td>f0</td><td>ZT4|control|SSO</td><td>ZT4|1mM OA|SSO</td><td>6.51E-64</td><td>4.40E-62</td><td>< 2e-16</td><td>****</td><td>Wilcoxon</td></tr>
<tr><td>f0</td><td>ZT4|control|SSO</td><td>ZT4|1mM OA|Quiescent</td><td>5.57E-32</td><td>2.80E-30</td><td>< 2e-16</td><td>****</td><td>Wilcoxon</td></tr>
<tr><td>f0</td><td>ZT4|control|SSO</td><td>ZT4|10mM OA|SSO</td><td>1.01E-20</td><td>3.70E-19</td><td>< 2e-16</td><td>****</td><td>Wilcoxon</td></tr>
<tr><td>f0</td><td>ZT4|control|SSO</td><td>ZT4|10mM OA|Quiescent</td><td>2.41E-51</td><td>1.50E-49</td><td>< 2e-16</td><td>****</td><td>Wilcoxon</td></tr>
<tr><td>f0</td><td>ZT4|control|SSO</td><td>ZT12|baseline|SSO</td><td>0.451272</td><td>1</td><td>0.45127</td><td>ns</td><td>Wilcoxon</td></tr>
<tr><td>f0</td><td>ZT4|control|SSO</td><td>ZT12|control|SSO</td><td>0.215733</td><td>1</td><td>0.21573</td><td>ns</td><td>Wilcoxon</td></tr>
<tr><td>f0</td><td>ZT4|control|SSO</td><td>ZT12|control|Quiescent</td><td>0.02566</td><td>0.38</td><td>0.02566</td><td>*</td><td>Wilcoxon</td></tr>
<tr><td>f0</td><td>ZT4|control|SSO</td><td>ZT12|1mM OA|SSO</td><td>1.20E-10</td><td>3.10E-09</td><td>1.20E-10</td><td>****</td><td>Wilcoxon</td></tr>
<tr><td>f0</td><td>ZT4|control|SSO</td><td>ZT12|1mM OA|Quiescent</td><td>3.19E-74</td><td>2.20E-72</td><td>< 2e-16</td><td>****</td><td>Wilcoxon</td></tr>
<tr><td>f0</td><td>ZT4|control|SSO</td><td>ZT12|10mM OA|SSO</td><td>8.18E-22</td><td>3.30E-20</td><td>< 2e-16</td><td>****</td><td>Wilcoxon</td></tr>
<tr><td>f0</td><td>ZT4|control|SSO</td><td>ZT12|10mM OA|Quiescent</td><td>1.72E-70</td><td>1.20E-68</td><td>< 2e-16</td><td>****</td><td>Wilcoxon</td></tr>
<tr><td>f0</td><td>ZT4|1mM OA|SSO</td><td>ZT4|1mM OA|Quiescent</td><td>0.413076</td><td>1</td><td>0.41308</td><td>ns</td><td>Wilcoxon</td></tr>
<tr><td>f0</td><td>ZT4|1mM OA|SSO</td><td>ZT4|10mM OA|SSO</td><td>1.17E-23</td><td>5.30E-22</td><td>< 2e-16</td><td>****</td><td>Wilcoxon</td></tr>
<tr><td>f0</td><td>ZT4|1mM OA|SSO</td><td>ZT4|10mM OA|Quiescent</td><td>7.90E-40</td><td>4.60E-38</td><td>< 2e-16</td><td>****</td><td>Wilcoxon</td></tr>
<tr><td>f0</td><td>ZT4|1mM OA|SSO</td><td>ZT12|baseline|SSO</td><td>2.35E-73</td><td>1.60E-71</td><td>< 2e-16</td><td>****</td><td>Wilcoxon</td></tr>
<tr><td>f0</td><td>ZT4|1mM OA|SSO</td><td>ZT12|control|SSO</td><td>2.99E-96</td><td>2.20E-94</td><td>< 2e-16</td><td>****</td><td>Wilcoxon</td></tr>
<tr><td>f0</td><td>ZT4|1mM OA|SSO</td><td>ZT12|control|Quiescent</td><td>1.96E-37</td><td>1.10E-35</td><td>< 2e-16</td><td>****</td><td>Wilcoxon</td></tr>
<tr><td>f0</td><td>ZT4|1mM OA|SSO</td><td>ZT12|1mM OA|SSO</td><td>9.58E-32</td><td>4.80E-30</td><td>< 2e-16</td><td>****</td><td>Wilcoxon</td></tr>
<tr><td>f0</td><td>ZT4|1mM OA|SSO</td><td>ZT12|1mM OA|Quiescent</td><td>0.018585</td><td>0.32</td><td>0.01858</td><td>*</td><td>Wilcoxon</td></tr>
<tr><td>f0</td><td>ZT4|1mM OA|SSO</td><td>ZT12|10mM OA|SSO</td><td>0.175406</td><td>1</td><td>0.17541</td><td>ns</td><td>Wilcoxon</td></tr>
<tr><td>f0</td><td>ZT4|1mM OA|SSO</td><td>ZT12|10mM OA|Quiescent</td><td>0.075395</td><td>0.98</td><td>0.07539</td><td>ns</td><td>Wilcoxon</td></tr>
<tr><td>f0</td><td>ZT4|1mM OA|Quiescent</td><td>ZT4|10mM OA|SSO</td><td>6.04E-08</td><td>1.50E-06</td><td>6.00E-08</td><td>****</td><td>Wilcoxon</td></tr>
<tr><td>f0</td><td>ZT4|1mM OA|Quiescent</td><td>ZT4|10mM OA|Quiescent</td><td>1.52E-07</td><td>3.70E-06</td><td>1.50E-07</td><td>****</td><td>Wilcoxon</td></tr>
<tr><td>f0</td><td>ZT4|1mM OA|Quiescent</td><td>ZT12|baseline|SSO</td><td>5.59E-38</td><td>3.10E-36</td><td>< 2e-16</td><td>****</td><td>Wilcoxon</td></tr>
<tr><td>f0</td><td>ZT4|1mM OA|Quiescent</td><td>ZT12|control|SSO</td><td>8.69E-41</td><td>5.20E-39</td><td>< 2e-16</td><td>****</td><td>Wilcoxon</td></tr>
</table>

f0	ZT4|1mM OA|Quiescent	ZT12|control|Quiescent	4.98E-20	1.80E-18	< 2e-16	****	Wilcoxon
f0	ZT4|1mM OA|Quiescent	ZT12|1mM OA|SSO	1.54E-16	4.50E-15	< 2e-16	****	Wilcoxon
f0	ZT4|1mM OA|Quiescent	ZT12|1mM OA|Quiescent	0.760695	1	0.76069	ns	Wilcoxon
f0	ZT4|1mM OA|Quiescent	ZT12|10mM OA|SSO	0.037447	0.52	0.03745	*	Wilcoxon
f0	ZT4|1mM OA|Quiescent	ZT12|10mM OA|Quiescent	0.77202	1	0.77202	ns	Wilcoxon
f0	ZT4|10mM OA|SSO	ZT4|10mM OA|Quiescent	3.12E-41	1.90E-39	< 2e-16	****	Wilcoxon
f0	ZT4|10mM OA|SSO	ZT12|baseline|SSO	8.20E-21	3.10E-19	< 2e-16	****	Wilcoxon
f0	ZT4|10mM OA|SSO	ZT12|control|SSO	8.97E-11	2.40E-09	9.00E-11	****	Wilcoxon
f0	ZT4|10mM OA|SSO	ZT12|control|Quiescent	0.000309	0.0059	0.00031	***	Wilcoxon
f0	ZT4|10mM OA|SSO	ZT12|1mM OA|SSO	0.017707	0.32	0.01771	*	Wilcoxon
f0	ZT4|10mM OA|SSO	ZT12|1mM OA|Quiescent	1.02E-28	4.80E-27	< 2e-16	****	Wilcoxon
f0	ZT4|10mM OA|SSO	ZT12|10mM OA|SSO	7.04E-07	1.60E-05	7.00E-07	****	Wilcoxon
f0	ZT4|10mM OA|SSO	ZT12|10mM OA|Quiescent	4.57E-29	2.20E-27	< 2e-16	****	Wilcoxon
f0	ZT4|10mM OA|Quiescent	ZT12|baseline|SSO	8.73E-53	5.60E-51	< 2e-16	****	Wilcoxon
f0	ZT4|10mM OA|Quiescent	ZT12|control|SSO	2.16E-61	1.40E-59	< 2e-16	****	Wilcoxon
f0	ZT4|10mM OA|Quiescent	ZT12|control|Quiescent	1.22E-37	6.70E-36	< 2e-16	****	Wilcoxon
f0	ZT4|10mM OA|Quiescent	ZT12|1mM OA|SSO	2.74E-34	1.40E-32	< 2e-16	****	Wilcoxon
f0	ZT4|10mM OA|Quiescent	ZT12|1mM OA|Quiescent	1.53E-29	7.50E-28	< 2e-16	****	Wilcoxon
f0	ZT4|10mM OA|Quiescent	ZT12|10mM OA|SSO	4.07E-06	9.00E-05	4.10E-06	****	Wilcoxon
f0	ZT4|10mM OA|Quiescent	ZT12|10mM OA|Quiescent	1.11E-18	3.60E-17	< 2e-16	****	Wilcoxon
f0	ZT12|baseline|SSO	ZT12|control|SSO	5.58E-05	0.0011	5.60E-05	****	Wilcoxon
f0	ZT12|baseline|SSO	ZT12|control|Quiescent	0.11584	1	0.11584	ns	Wilcoxon
f0	ZT12|baseline|SSO	ZT12|1mM OA|SSO	1.14E-16	3.40E-15	< 2e-16	****	Wilcoxon
f0	ZT12|baseline|SSO	ZT12|1mM OA|Quiescent	3.03E-80	2.20E-78	< 2e-16	****	Wilcoxon
f0	ZT12|baseline|SSO	ZT12|10mM OA|SSO	4.54E-22	1.90E-20	< 2e-16	****	Wilcoxon

f0	ZT12|baseline|SSO	ZT12|10mM OA|Quiescent	8.57E-83	6.20E-81	< 2e-16	****	Wilcoxon
f0	ZT12|control|SSO	ZT12|control|Quiescent	1.57E-22	6.60E-21	< 2e-16	****	Wilcoxon
f0	ZT12|control|SSO	ZT12|1mM OA|SSO	3.79E-19	1.30E-17	< 2e-16	****	Wilcoxon
f0	ZT12|control|SSO	ZT12|1mM OA|Quiescent	1.70E-110	1.30E-108	< 2e-16	****	Wilcoxon
f0	ZT12|control|SSO	ZT12|10mM OA|SSO	1.09E-22	4.70E-21	< 2e-16	****	Wilcoxon
f0	ZT12|control|SSO	ZT12|10mM OA|Quiescent	1.80E-130	1.40E-128	< 2e-16	****	Wilcoxon
f0	ZT12|control|Quiescent	ZT12|1mM OA|SSO	2.80E-15	7.80E-14	2.80E-15	****	Wilcoxon
f0	ZT12|control|Quiescent	ZT12|1mM OA|Quiescent	7.12E-39	4.10E-37	< 2e-16	****	Wilcoxon
f0	ZT12|control|Quiescent	ZT12|10mM OA|SSO	6.86E-18	2.20E-16	< 2e-16	****	Wilcoxon
f0	ZT12|control|Quiescent	ZT12|10mM OA|Quiescent	7.97E-43	4.90E-41	< 2e-16	****	Wilcoxon
f0	ZT12|1mM OA|SSO	ZT12|1mM OA|Quiescent	1.49E-36	7.90E-35	< 2e-16	****	Wilcoxon
f0	ZT12|1mM OA|SSO	ZT12|10mM OA|SSO	1.31E-17	4.10E-16	< 2e-16	****	Wilcoxon
f0	ZT12|1mM OA|SSO	ZT12|10mM OA|Quiescent	1.54E-25	7.10E-24	< 2e-16	****	Wilcoxon
f0	ZT12|1mM OA|Quiescent	ZT12|10mM OA|SSO	0.325328	1	0.32533	ns	Wilcoxon
f0	ZT12|1mM OA|Quiescent	ZT12|10mM OA|Quiescent	0.754713	1	0.75471	ns	Wilcoxon
f0	ZT12|10mM OA|SSO	ZT12|10mM OA|Quiescent	0.664616	1	0.66462	ns	Wilcoxon
peak	ZT4|baseline|SSO	ZT4|control|SSO	0.638558	1	0.63856	ns	Wilcoxon
peak	ZT4|baseline|SSO	ZT4|1mM OA|SSO	6.96E-85	5.10E-83	< 2e-16	****	Wilcoxon
peak	ZT4|baseline|SSO	ZT4|1mM OA|Quiescent	2.49E-40	1.50E-38	< 2e-16	****	Wilcoxon
peak	ZT4|baseline|SSO	ZT4|10mM OA|SSO	2.59E-21	1.00E-19	< 2e-16	****	Wilcoxon
peak	ZT4|baseline|SSO	ZT4|10mM OA|Quiescent	2.93E-57	1.90E-55	< 2e-16	****	Wilcoxon
peak	ZT4|baseline|SSO	ZT12|baseline|SSO	0.097019	1	0.09702	ns	Wilcoxon
peak	ZT4|baseline|SSO	ZT12|control|SSO	1.10E-05	0.00023	1.10E-05	****	Wilcoxon
peak	ZT4|baseline|SSO	ZT12|control|Quiescent	0.019414	0.32	0.01941	*	Wilcoxon
peak	ZT4|baseline|SSO	ZT12|1mM OA|SSO	4.22E-19	1.40E-17	< 2e-16	****	Wilcoxon

peak	ZT4|baseline|SSO	ZT12|1mM OA|Quiescent	2.35E-97	1.80E-95	< 2e-16	****	Wilcoxon
peak	ZT4|baseline|SSO	ZT12|10mM OA|SSO	9.89E-23	4.40E-21	< 2e-16	****	Wilcoxon
peak	ZT4|baseline|SSO	ZT12|10mM OA|Quiescent	1.51E-103	1.10E-101	< 2e-16	****	Wilcoxon
peak	ZT4|control|SSO	ZT4|1mM OA|SSO	6.51E-64	4.40E-62	< 2e-16	****	Wilcoxon
peak	ZT4|control|SSO	ZT4|1mM OA|Quiescent	5.57E-32	2.80E-30	< 2e-16	****	Wilcoxon
peak	ZT4|control|SSO	ZT4|10mM OA|SSO	1.01E-20	3.70E-19	< 2e-16	****	Wilcoxon
peak	ZT4|control|SSO	ZT4|10mM OA|Quiescent	2.41E-51	1.50E-49	< 2e-16	****	Wilcoxon
peak	ZT4|control|SSO	ZT12|baseline|SSO	0.451272	1	0.45127	ns	Wilcoxon
peak	ZT4|control|SSO	ZT12|control|SSO	0.215733	1	0.21573	ns	Wilcoxon
peak	ZT4|control|SSO	ZT12|control|Quiescent	0.02566	0.38	0.02566	*	Wilcoxon
peak	ZT4|control|SSO	ZT12|1mM OA|SSO	1.20E-10	3.10E-09	1.20E-10	****	Wilcoxon
peak	ZT4|control|SSO	ZT12|1mM OA|Quiescent	3.19E-74	2.20E-72	< 2e-16	****	Wilcoxon
peak	ZT4|control|SSO	ZT12|10mM OA|SSO	8.18E-22	3.30E-20	< 2e-16	****	Wilcoxon
peak	ZT4|control|SSO	ZT12|10mM OA|Quiescent	1.72E-70	1.20E-68	< 2e-16	****	Wilcoxon
peak	ZT4|1mM OA|SSO	ZT4|1mM OA|Quiescent	0.413076	1	0.41308	ns	Wilcoxon
peak	ZT4|1mM OA|SSO	ZT4|10mM OA|SSO	1.17E-23	5.30E-22	< 2e-16	****	Wilcoxon
peak	ZT4|1mM OA|SSO	ZT4|10mM OA|Quiescent	7.90E-40	4.60E-38	< 2e-16	****	Wilcoxon
peak	ZT4|1mM OA|SSO	ZT12|baseline|SSO	2.35E-73	1.60E-71	< 2e-16	****	Wilcoxon
peak	ZT4|1mM OA|SSO	ZT12|control|SSO	2.99E-96	2.20E-94	< 2e-16	****	Wilcoxon
peak	ZT4|1mM OA|SSO	ZT12|control|Quiescent	1.96E-37	1.10E-35	< 2e-16	****	Wilcoxon
peak	ZT4|1mM OA|SSO	ZT12|1mM OA|SSO	9.58E-32	4.80E-30	< 2e-16	****	Wilcoxon
peak	ZT4|1mM OA|SSO	ZT12|1mM OA|Quiescent	0.018585	0.32	0.01858	*	Wilcoxon
peak	ZT4|1mM OA|SSO	ZT12|10mM OA|SSO	0.175406	1	0.17541	ns	Wilcoxon
peak	ZT4|1mM OA|SSO	ZT12|10mM OA|Quiescent	0.075395	0.98	0.07539	ns	Wilcoxon
peak	ZT4|1mM OA|Quiescent	ZT4|10mM OA|SSO	6.04E-08	1.50E-06	6.00E-08	****	Wilcoxon

peak	ZT4|1mM OA|Quiescent	ZT4|10mM OA|Quiescent	1.52E-07	3.70E-06	1.50E-07	****	Wilcoxon
peak	ZT4|1mM OA|Quiescent	ZT12|baseline|SSO	5.59E-38	3.10E-36	< 2e-16	****	Wilcoxon
peak	ZT4|1mM OA|Quiescent	ZT12|control|SSO	8.69E-41	5.20E-39	< 2e-16	****	Wilcoxon
peak	ZT4|1mM OA|Quiescent	ZT12|control|Quiescent	4.98E-20	1.80E-18	< 2e-16	****	Wilcoxon
peak	ZT4|1mM OA|Quiescent	ZT12|1mM OA|SSO	1.54E-16	4.50E-15	< 2e-16	****	Wilcoxon
peak	ZT4|1mM OA|Quiescent	ZT12|1mM OA|Quiescent	0.760695	1	0.76069	ns	Wilcoxon
peak	ZT4|1mM OA|Quiescent	ZT12|10mM OA|SSO	0.037447	0.52	0.03745	*	Wilcoxon
peak	ZT4|1mM OA|Quiescent	ZT12|10mM OA|Quiescent	0.77202	1	0.77202	ns	Wilcoxon
peak	ZT4|10mM OA|SSO	ZT4|10mM OA|Quiescent	3.12E-41	1.90E-39	< 2e-16	****	Wilcoxon
peak	ZT4|10mM OA|SSO	ZT12|baseline|SSO	8.20E-21	3.10E-19	< 2e-16	****	Wilcoxon
peak	ZT4|10mM OA|SSO	ZT12|control|SSO	8.97E-11	2.40E-09	9.00E-11	****	Wilcoxon
peak	ZT4|10mM OA|SSO	ZT12|control|Quiescent	0.000309	0.0059	0.00031	***	Wilcoxon
peak	ZT4|10mM OA|SSO	ZT12|1mM OA|SSO	0.017707	0.32	0.01771	*	Wilcoxon
peak	ZT4|10mM OA|SSO	ZT12|1mM OA|Quiescent	1.02E-28	4.80E-27	< 2e-16	****	Wilcoxon
peak	ZT4|10mM OA|SSO	ZT12|10mM OA|SSO	7.04E-07	1.60E-05	7.00E-07	****	Wilcoxon
peak	ZT4|10mM OA|SSO	ZT12|10mM OA|Quiescent	4.57E-29	2.20E-27	< 2e-16	****	Wilcoxon
peak	ZT4|10mM OA|Quiescent	ZT12|baseline|SSO	8.73E-53	5.60E-51	< 2e-16	****	Wilcoxon
peak	ZT4|10mM OA|Quiescent	ZT12|control|SSO	2.16E-61	1.40E-59	< 2e-16	****	Wilcoxon
peak	ZT4|10mM OA|Quiescent	ZT12|control|Quiescent	1.22E-37	6.70E-36	< 2e-16	****	Wilcoxon
peak	ZT4|10mM OA|Quiescent	ZT12|1mM OA|SSO	2.74E-34	1.40E-32	< 2e-16	****	Wilcoxon
peak	ZT4|10mM OA|Quiescent	ZT12|1mM OA|Quiescent	1.53E-29	7.50E-28	< 2e-16	****	Wilcoxon
peak	ZT4|10mM OA|Quiescent	ZT12|10mM OA|SSO	4.07E-06	9.00E-05	4.10E-06	****	Wilcoxon
peak	ZT4|10mM OA|Quiescent	ZT12|10mM OA|Quiescent	1.11E-18	3.60E-17	< 2e-16	****	Wilcoxon
peak	ZT12|baseline|SSO	ZT12|control|SSO	5.58E-05	0.0011	5.60E-05	****	Wilcoxon
peak	ZT12|baseline|SSO	ZT12|control|Quiescent	0.11584	1	0.11584	ns	Wilcoxon

peak	ZT12|baseline|SSO	ZT12|1mM OA|SSO	1.14E-16	3.40E-15	< 2e-16	****	Wilcoxon
peak	ZT12|baseline|SSO	ZT12|1mM OA|Quiescent	3.03E-80	2.20E-78	< 2e-16	****	Wilcoxon
peak	ZT12|baseline|SSO	ZT12|10mM OA|SSO	4.54E-22	1.90E-20	< 2e-16	****	Wilcoxon
peak	ZT12|baseline|SSO	ZT12|10mM OA|Quiescent	8.57E-83	6.20E-81	< 2e-16	****	Wilcoxon
peak	ZT12|control|SSO	ZT12|control|Quiescent	1.57E-22	6.60E-21	< 2e-16	****	Wilcoxon
peak	ZT12|control|SSO	ZT12|1mM OA|SSO	3.79E-19	1.30E-17	< 2e-16	****	Wilcoxon
peak	ZT12|control|SSO	ZT12|1mM OA|Quiescent	1.70E-110	1.30E-108	< 2e-16	****	Wilcoxon
peak	ZT12|control|SSO	ZT12|10mM OA|SSO	1.09E-22	4.70E-21	< 2e-16	****	Wilcoxon
peak	ZT12|control|SSO	ZT12|10mM OA|Quiescent	1.80E-130	1.40E-128	< 2e-16	****	Wilcoxon
peak	ZT12|control|Quiescent	ZT12|1mM OA|SSO	2.80E-15	7.80E-14	2.80E-15	****	Wilcoxon
peak	ZT12|control|Quiescent	ZT12|1mM OA|Quiescent	7.12E-39	4.10E-37	< 2e-16	****	Wilcoxon
peak	ZT12|control|Quiescent	ZT12|10mM OA|SSO	6.86E-18	2.20E-16	< 2e-16	****	Wilcoxon
peak	ZT12|control|Quiescent	ZT12|10mM OA|Quiescent	7.97E-43	4.90E-41	< 2e-16	****	Wilcoxon
peak	ZT12|1mM OA|SSO	ZT12|1mM OA|Quiescent	1.49E-36	7.90E-35	< 2e-16	****	Wilcoxon
peak	ZT12|1mM OA|SSO	ZT12|10mM OA|SSO	1.31E-17	4.10E-16	< 2e-16	****	Wilcoxon
peak	ZT12|1mM OA|SSO	ZT12|10mM OA|Quiescent	1.54E-25	7.10E-24	< 2e-16	****	Wilcoxon
peak	ZT12|1mM OA|Quiescent	ZT12|10mM OA|SSO	0.325328	1	0.32533	ns	Wilcoxon
peak	ZT12|1mM OA|Quiescent	ZT12|10mM OA|Quiescent	0.754713	1	0.75471	ns	Wilcoxon
peak	ZT12|10mM OA|SSO	ZT12|10mM OA|Quiescent	0.664616	1	0.66462	ns	Wilcoxon
Q	ZT4|baseline|SSO	ZT4|control|SSO	0.495053	1	0.49505	ns	Wilcoxon
Q	ZT4|baseline|SSO	ZT4|1mM OA|SSO	2.25E-23	1.50E-21	< 2e-16	****	Wilcoxon
Q	ZT4|baseline|SSO	ZT4|1mM OA|Quiescent	1.57E-15	7.50E-14	1.60E-15	****	Wilcoxon
Q	ZT4|baseline|SSO	ZT4|10mM OA|SSO	0.470877	1	0.47088	ns	Wilcoxon
Q	ZT4|baseline|SSO	ZT4|10mM OA|Quiescent	3.27E-18	1.70E-16	< 2e-16	****	Wilcoxon
Q	ZT4|baseline|SSO	ZT12|baseline|SSO	0.030219	0.27	0.03022	*	Wilcoxon

Q	ZT4|baseline|SSO	ZT12|control|SSO	0.0058 42	0.093	0.0058 4	**	Wilcox on
Q	ZT4|baseline|SSO	ZT12|control|Quie scent	7.43E-27	5.10E-25	< 2e-16	****	Wilcox on
Q	ZT4|baseline|SSO	ZT12|1mM OA|SSO	2.96E-08	1.10E-06	3.00E-08	****	Wilcox on
Q	ZT4|baseline|SSO	ZT12|1mM OA|Quiescent	2.39E-18	1.30E-16	< 2e-16	****	Wilcox on
Q	ZT4|baseline|SSO	ZT12|10mM OA|SSO	0.0009 43	0.02	0.0009 4	***	Wilcox on
Q	ZT4|baseline|SSO	ZT12|10mM OA|Quiescent	5.69E-71	4.40E-69	< 2e-16	****	Wilcox on
Q	ZT4|control|SSO	ZT4|1mM OA|SSO	1.26E-26	8.40E-25	< 2e-16	****	Wilcox on
Q	ZT4|control|SSO	ZT4|1mM OA|Quiescent	4.70E-14	2.20E-12	4.70E-14	****	Wilcox on
Q	ZT4|control|SSO	ZT4|10mM OA|SSO	0.0061 23	0.093	0.0061 2	**	Wilcox on
Q	ZT4|control|SSO	ZT4|10mM OA|Quiescent	5.22E-14	2.30E-12	5.20E-14	****	Wilcox on
Q	ZT4|control|SSO	ZT12|baseline|SSO	0.3544 27	1	0.3544 3	ns	Wilcox on
Q	ZT4|control|SSO	ZT12|control|SSO	0.5586 53	1	0.5586 5	ns	Wilcox on
Q	ZT4|control|SSO	ZT12|control|Quie scent	1.05E-05	0.000 32	1.10E-05	****	Wilcox on
Q	ZT4|control|SSO	ZT12|1mM OA|SSO	9.20E-05	0.002 3	9.20E-05	****	Wilcox on
Q	ZT4|control|SSO	ZT12|1mM OA|Quiescent	7.90E-11	3.50E-09	7.90E-11	****	Wilcox on
Q	ZT4|control|SSO	ZT12|10mM OA|SSO	0.3366 58	1	0.3366 6	ns	Wilcox on
Q	ZT4|control|SSO	ZT12|10mM OA|Quiescent	4.46E-54	3.30E-52	< 2e-16	****	Wilcox on
Q	ZT4|1mM OA|SSO	ZT4|1mM OA|Quiescent	5.45E-23	3.50E-21	< 2e-16	****	Wilcox on
Q	ZT4|1mM OA|SSO	ZT4|10mM OA|SSO	8.12E-16	4.00E-14	8.10E-16	****	Wilcox on
Q	ZT4|1mM OA|SSO	ZT4|10mM OA|Quiescent	3.65E-21	2.20E-19	< 2e-16	****	Wilcox on
Q	ZT4|1mM OA|SSO	ZT12|baseline|SSO	8.49E-19	4.80E-17	< 2e-16	****	Wilcox on
Q	ZT4|1mM OA|SSO	ZT12|control|SSO	6.56E-31	4.60E-29	< 2e-16	****	Wilcox on
Q	ZT4|1mM OA|SSO	ZT12|control|Quie scent	4.00E-35	2.90E-33	< 2e-16	****	Wilcox on
Q	ZT4|1mM OA|SSO	ZT12|1mM OA|SSO	1.92E-20	1.20E-18	< 2e-16	****	Wilcox on
Q	ZT4|1mM OA|SSO	ZT12|1mM OA|Quiescent	1.12E-17	5.70E-16	< 2e-16	****	Wilcox on

Q	ZT4|1mM OA|SSO	ZT12|10mM OA|SSO	3.92E-08	1.40E-06	3.90E-08	****	Wilcoxon
Q	ZT4|1mM OA|SSO	ZT12|10mM OA|Quiescent	2.29E-53	1.70E-51	< 2e-16	****	Wilcoxon
Q	ZT4|1mM OA|Quiescent	ZT4|10mM OA|SSO	2.91E-16	1.50E-14	2.90E-16	****	Wilcoxon
Q	ZT4|1mM OA|Quiescent	ZT4|10mM OA|Quiescent	0.881944	1	0.88194	ns	Wilcoxon
Q	ZT4|1mM OA|Quiescent	ZT12|baseline|SSO	4.41E-10	1.80E-08	4.40E-10	****	Wilcoxon
Q	ZT4|1mM OA|Quiescent	ZT12|control|SSO	2.14E-15	1.00E-13	2.10E-15	****	Wilcoxon
Q	ZT4|1mM OA|Quiescent	ZT12|control|Quiescent	3.83E-07	1.30E-05	3.80E-07	****	Wilcoxon
Q	ZT4|1mM OA|Quiescent	ZT12|1mM OA|SSO	0.001347	0.027	0.00135	**	Wilcoxon
Q	ZT4|1mM OA|Quiescent	ZT12|1mM OA|Quiescent	0.007616	0.099	0.00762	**	Wilcoxon
Q	ZT4|1mM OA|Quiescent	ZT12|10mM OA|SSO	0.00282	0.051	0.00282	**	Wilcoxon
Q	ZT4|1mM OA|Quiescent	ZT12|10mM OA|Quiescent	1.96E-05	0.00053	2.00E-05	****	Wilcoxon
Q	ZT4|10mM OA|SSO	ZT4|10mM OA|Quiescent	1.47E-18	8.10E-17	< 2e-16	****	Wilcoxon
Q	ZT4|10mM OA|SSO	ZT12|baseline|SSO	0.621794	1	0.62179	ns	Wilcoxon
Q	ZT4|10mM OA|SSO	ZT12|control|SSO	0.000771	0.017	0.00077	***	Wilcoxon
Q	ZT4|10mM OA|SSO	ZT12|control|Quiescent	1.48E-33	1.00E-31	< 2e-16	****	Wilcoxon
Q	ZT4|10mM OA|SSO	ZT12|1mM OA|SSO	2.93E-09	1.10E-07	2.90E-09	****	Wilcoxon
Q	ZT4|10mM OA|SSO	ZT12|1mM OA|Quiescent	3.58E-20	2.10E-18	< 2e-16	****	Wilcoxon
Q	ZT4|10mM OA|SSO	ZT12|10mM OA|SSO	1.19E-05	0.00033	1.20E-05	****	Wilcoxon
Q	ZT4|10mM OA|SSO	ZT12|10mM OA|Quiescent	1.21E-59	9.20E-58	< 2e-16	****	Wilcoxon
Q	ZT4|10mM OA|Quiescent	ZT12|baseline|SSO	1.26E-10	5.40E-09	1.30E-10	****	Wilcoxon
Q	ZT4|10mM OA|Quiescent	ZT12|control|SSO	1.70E-18	9.20E-17	< 2e-16	****	Wilcoxon
Q	ZT4|10mM OA|Quiescent	ZT12|control|Quiescent	7.89E-07	2.50E-05	7.90E-07	****	Wilcoxon
Q	ZT4|10mM OA|Quiescent	ZT12|1mM OA|SSO	0.000231	0.0056	0.00023	***	Wilcoxon
Q	ZT4|10mM OA|Quiescent	ZT12|1mM OA|Quiescent	0.023009	0.23	0.02301	*	Wilcoxon
Q	ZT4|10mM OA|Quiescent	ZT12|10mM OA|SSO	0.001984	0.038	0.00198	**	Wilcoxon

Q	ZT4|10mM OA|Quiescent	ZT12|10mM OA|Quiescent	7.60E-08	2.70E-06	7.60E-08	****	Wilcoxon
Q	ZT12|baseline|SSO	ZT12|control|SSO	4.47E-07	1.50E-05	4.50E-07	****	Wilcoxon
Q	ZT12|baseline|SSO	ZT12|control|Quiescent	2.48E-20	1.50E-18	< 2e-16	****	Wilcoxon
Q	ZT12|baseline|SSO	ZT12|1mM OA|SSO	1.07E-05	0.00032	1.10E-05	****	Wilcoxon
Q	ZT12|baseline|SSO	ZT12|1mM OA|Quiescent	3.58E-10	1.50E-08	3.60E-10	****	Wilcoxon
Q	ZT12|baseline|SSO	ZT12|10mM OA|SSO	0.006734	0.094	0.00673	**	Wilcoxon
Q	ZT12|baseline|SSO	ZT12|10mM OA|Quiescent	2.04E-47	1.50E-45	< 2e-16	****	Wilcoxon
Q	ZT12|control|SSO	ZT12|control|Quiescent	6.94E-20	4.00E-18	< 2e-16	****	Wilcoxon
Q	ZT12|control|SSO	ZT12|1mM OA|SSO	5.40E-06	0.00017	5.40E-06	****	Wilcoxon
Q	ZT12|control|SSO	ZT12|1mM OA|Quiescent	1.44E-22	9.00E-21	< 2e-16	****	Wilcoxon
Q	ZT12|control|SSO	ZT12|10mM OA|SSO	0.003177	0.054	0.00318	**	Wilcoxon
Q	ZT12|control|SSO	ZT12|10mM OA|Quiescent	2.87E-89	2.20E-87	< 2e-16	****	Wilcoxon
Q	ZT12|control|Quiescent	ZT12|1mM OA|SSO	0.000466	0.011	0.00047	***	Wilcoxon
Q	ZT12|control|Quiescent	ZT12|1mM OA|Quiescent	2.73E-08	1.00E-06	2.70E-08	****	Wilcoxon
Q	ZT12|control|Quiescent	ZT12|10mM OA|SSO	3.22E-05	0.00084	3.20E-05	****	Wilcoxon
Q	ZT12|control|Quiescent	ZT12|10mM OA|Quiescent	1.21E-26	8.20E-25	< 2e-16	****	Wilcoxon
Q	ZT12|1mM OA|SSO	ZT12|1mM OA|Quiescent	0.018242	0.21	0.01824	*	Wilcoxon
Q	ZT12|1mM OA|SSO	ZT12|10mM OA|SSO	0.587354	1	0.58735	ns	Wilcoxon
Q	ZT12|1mM OA|SSO	ZT12|10mM OA|Quiescent	3.27E-21	2.00E-19	< 2e-16	****	Wilcoxon
Q	ZT12|1mM OA|Quiescent	ZT12|10mM OA|SSO	0.01716	0.21	0.01716	*	Wilcoxon
Q	ZT12|1mM OA|Quiescent	ZT12|10mM OA|Quiescent	3.58E-24	2.40E-22	< 2e-16	****	Wilcoxon
Q	ZT12|10mM OA|SSO	ZT12|10mM OA|Quiescent	1.58E-09	6.30E-08	1.60E-09	****	Wilcoxon

Supplementary table 5: Summary of steady-state stiffness values extracted from force-step stimulation responses in wildtype male mosquitoes upon exposure to octopamine

condition	parameters	mean	sd	median	mad
ZT4_G3_baseline	Steady-state stiffness	189.8061	42.64113	172.7202	35.92604
ZT4_G3_Control	Steady-state stiffness	222.3794	129.4798	181.992	88.48203
ZT4_G3_OA1	Steady-state stiffness	456.8191	364.7843	332.2196	196.1043
ZT4_G3_OA10	Steady-state stiffness	531.0592	473.7489	329.3449	218.8008
ZT12_G3_baseline	Steady-state stiffness	209.6827	80.47488	185.1286	34.68105
ZT12_G3_Control	Steady-state stiffness	337.7086	335.975	194.562	65.67948
ZT12_G3_OA1	Steady-state stiffness	488.5785	366.961	395.9188	280.4134
ZT12_G3_OA10	Steady-state stiffness	1442.868	868.1435	1257.664	682.8566

Supplementary table 6: Two-sided wilcoxon signed-rank test on force-step stimulation analysis in males (pairwise comparisons). The Holms procedure was used to correct for multiple comparisons.

group1	group2	p	p.adj	p.format	p.signif	method
ZT04|baseline	ZT12|baseline	0.222254	1	0.22225	ns	Wilcoxon
ZT04|baseline	ZT04|control	0.890034	1	0.89003	ns	Wilcoxon
ZT04|baseline	ZT12|control	0.258832	1	0.25883	ns	Wilcoxon
ZT04|baseline	ZT04|OA1mM	0.000128	0.0032	0.00013	***	Wilcoxon
ZT04|baseline	ZT12|OA1mM	0.001419	0.031	0.00142	**	Wilcoxon
ZT04|baseline	ZT04|OA10mM	0.010254	0.17	0.01025	*	Wilcoxon
ZT04|baseline	ZT12|OA10mM	1.81E-10	5.10E-09	1.80E-10	****	Wilcoxon
ZT12|baseline	ZT04|control	0.834392	1	0.83439	ns	Wilcoxon
ZT12|baseline	ZT12|control	0.664123	1	0.66412	ns	Wilcoxon
ZT12|baseline	ZT04|OA1mM	0.001544	0.032	0.00154	**	Wilcoxon
ZT12|baseline	ZT12|OA1mM	0.005651	0.11	0.00565	**	Wilcoxon
ZT12|baseline	ZT04|OA10mM	0.029921	0.45	0.02992	*	Wilcoxon
ZT12|baseline	ZT12|OA10mM	7.70E-07	2.10E-05	7.70E-07	****	Wilcoxon
ZT04|control	ZT12|control	0.393048	1	0.39305	ns	Wilcoxon
ZT04|control	ZT04|OA1mM	0.024916	0.4	0.02492	*	Wilcoxon
ZT04|control	ZT12|OA1mM	0.043421	0.61	0.04342	*	Wilcoxon
ZT04|control	ZT04|OA10mM	0.052426	0.68	0.05243	ns	Wilcoxon
ZT04|control	ZT12|OA10mM	6.19E-06	0.00016	6.20E-06	****	Wilcoxon

ZT12|control	ZT04|OA1mM	0.122911	1	0.12291	ns	Wilcoxon
ZT12|control	ZT12|OA1mM	0.14571	1	0.14571	ns	Wilcoxon
ZT12|control	ZT04|OA10mM	0.247451	1	0.24745	ns	Wilcoxon
ZT12|control	ZT12|OA10mM	0.000473	0.011	0.00047	***	Wilcoxon
ZT04|OA1mM	ZT12|OA1mM	0.792077	1	0.79208	ns	Wilcoxon
ZT04|OA1mM	ZT04|OA10mM	0.821204	1	0.8212	ns	Wilcoxon
ZT04|OA1mM	ZT12|OA10mM	0.000496	0.011	0.0005	***	Wilcoxon
ZT12|OA1mM	ZT04|OA10mM	0.828557	1	0.82856	ns	Wilcoxon
ZT12|OA1mM	ZT12|OA10mM	0.002159	0.043	0.00216	**	Wilcoxon
ZT04|OA10mM	ZT12|OA10mM	0.005645	0.11	0.00564	**	Wilcoxon

Supplementary table 7: Summary of biophysical parameters extracted from frequency-modulated sweep responses in wildtype female mosquitoes upon exposure to octopamine

condition	parameters	mean	sd	median	mad
ZT4_G3_Ringer2	F0m	28.09141	10.57646	22.14493	6.319945
ZT4_G3_Ringer2	F0	331.3676	25.92163	330.5037	15.95556
ZT4_G3_Ringer2	peak	423.5824	43.12576	419.5956	45.48858
ZT4_G3_Ringer2	Q-factor	1.162511	0.110034	1.166847	0.124102
ZT4_G3_OA1	F0m	26.89788	8.827836	25.52511	9.233162
ZT4_G3_OA1	F0	325.3265	36.00657	311.4013	17.64739
ZT4_G3_OA1	peak	400.9052	65.95603	382.7119	35.90312
ZT4_G3_OA1	Q-factor	1.253819	0.132682	1.265837	0.12217
ZT12_G3_Ringer2	F0m	29.44617	12.26527	26.81448	13.38637
ZT12_G3_Ringer2	F0	388.9516	85.66718	343.4757	13.75166
ZT12_G3_Ringer2	peak	473.8941	93.9069	420.9028	36.08372
ZT12_G3_Ringer2	Q-factor	1.241297	0.086451	1.250919	0.102591
ZT12_G3_OA1	F0m	40.33739	21.3275	31.08491	7.541124
ZT12_G3_OA1	F0	441.7661	138.5823	351.7447	63.75718
ZT12_G3_OA1	peak	558.1338	169.2245	489.2491	155.8558
ZT12_G3_OA1	Q-factor	1.252156	0.599711	1.145468	0.167211

Supplementary table 8: Two-sided wilcoxon signed-rank test on frequency-modulated sweep analysis in females (pairwise comparisons). The Holms procedure was used to correct for multiple comparisons.

parameter	group1	group2	p	p.adj	p.format	p.signif	method
F0m	ZT4|Ringer 2	ZT4|1mM OA	0.981922	0.98	0.98	ns	Wilcoxon
F0m	ZT4|Ringer 2	ZT12|Ringer 2	0.428129	0.86	0.43	ns	Wilcoxon
F0m	ZT4|Ringer 2	ZT12|1mM OA	3.36E-27	2.00E-26	<2e-16	****	Wilcoxon
F0m	ZT4|1mM OA	ZT12|Ringer 2	0.185775	0.56	0.19	ns	Wilcoxon
F0m	ZT4|1mM OA	ZT12|1mM OA	3.16E-24	1.60E-23	<2e-16	****	Wilcoxon
F0m	ZT12|Ringer 2	ZT12|1mM OA	9.27E-18	3.70E-17	<2e-16	****	Wilcoxon
f0	ZT4|Ringer 2	ZT4|1mM OA	3.15E-10	6.30E-10	3.20E-10	****	Wilcoxon
f0	ZT4|Ringer 2	ZT12|Ringer 2	1.10E-42	6.60E-42	< 2e-16	****	Wilcoxon
f0	ZT4|Ringer 2	ZT12|1mM OA	7.83E-17	2.30E-16	< 2e-16	****	Wilcoxon
f0	ZT4|1mM OA	ZT12|Ringer 2	2.02E-42	1.00E-41	< 2e-16	****	Wilcoxon
f0	ZT4|1mM OA	ZT12|1mM OA	1.75E-40	7.00E-40	< 2e-16	****	Wilcoxon

f0	ZT12|Ringer 2	ZT12|1mM OA	0.169085	0.17	0.17	ns	Wilcoxon
peak	ZT4|Ringer 2	ZT4|1mM OA	9.54E-21	2.90E-20	< 2e-16	****	Wilcoxon
peak	ZT4|Ringer 2	ZT12|Ringer 2	9.90E-10	2.00E-09	9.90E-10	****	Wilcoxon
peak	ZT4|Ringer 2	ZT12|1mM OA	1.45E-23	5.80E-23	< 2e-16	****	Wilcoxon
peak	ZT4|1mM OA	ZT12|Ringer 2	8.56E-47	4.30E-46	< 2e-16	****	Wilcoxon
peak	ZT4|1mM OA	ZT12|1mM OA	5.08E-59	3.00E-58	< 2e-16	****	Wilcoxon
peak	ZT12|Ringer 2	ZT12|1mM OA	5.67E-05	5.70E-05	5.70E-05	****	Wilcoxon
Q	ZT4|Ringer 2	ZT4|1mM OA	8.25E-27	4.90E-26	< 2e-16	****	Wilcoxon
Q	ZT4|Ringer 2	ZT12|Ringer 2	1.20E-24	6.00E-24	< 2e-16	****	Wilcoxon
Q	ZT4|Ringer 2	ZT12|1mM OA	0.000643	0.0026	0.00064	***	Wilcoxon
Q	ZT4|1mM OA	ZT12|Ringer 2	0.400995	0.4	0.401	ns	Wilcoxon
Q	ZT4|1mM OA	ZT12|1mM OA	0.00138	0.0041	0.00138	**	Wilcoxon
Q	ZT12|Ringer 2	ZT12|1mM OA	0.002154	0.0043	0.00215	**	Wilcoxon

Supplementary table 9: Summary of steady-state stiffness values extracted from force-step stimulation responses in wildtype female mosquitoes upon exposure to octopamine

condition	parameters	mean	sd	median	mad
ZT4_G3_Ringer2	Steady-state stiffness	91.70988	15.74046	92.88549	18.0509
ZT4_G3_OA1	Steady-state stiffness	117.3927	13.22724	116.227	20.66096
ZT12_G3_Ringer2	Steady-state stiffness	144.9563	25.04838	144.3444	29.99264
ZT12_G3_OA1	Steady-state stiffness	140.7325	83.51008	116.654	16.96317

Supplementary table 10: Two-sided wilcoxon signed-rank test on force-step stimulation analysis in females (pairwise comparisons). The Holms procedure was used to correct for multiple comparisons.

parameter	group1	group2	p	p.adj	p.format	p.signif	method
value	ZT4|ringer2	ZT4|OA1mM	0.006993	0.035	0.007	**	Wilcoxon
value	ZT4|ringer2	ZT12|ringer2	0.00404	0.024	0.004	**	Wilcoxon

value	ZT4|ringer2	ZT12|OA1mM	0.037918	0.15	0.038	*	Wilcoxon
value	ZT4|OA1mM	ZT12|ringer2	0.153535	0.46	0.154	ns	Wilcoxon
value	ZT4|OA1mM	ZT12|OA1mM	0.878477	0.88	0.878	ns	Wilcoxon
value	ZT12|ringer2	ZT12|OA1mM	0.214141	0.46	0.214	ns	Wilcoxon

Supplementary table 11: Summary of biophysical parameters extracted from antennal free fluctuations of SSO flagella in octopamine receptor mutant male mosquitoes upon exposure to octopamine.

condition	State	parameter	mean	sd	median	mad
ZT12_G3_baseline	SSO	Frequency	357.7283	32.12737	358.5815	11.31134
ZT12_G3_baseline	SSO	Amplitude	383.7922	209.1748	386.9927	256.7351
ZT12_45_baseline	SSO	Frequency	358.5815	28.13053	353.241	7.917938
ZT12_45_baseline	SSO	Amplitude	762.3321	252.2291	793.5574	286.7445
ZT12_2886_baseline	SSO	Frequency	380.9368	27.80382	372.3145	11.31134
ZT12_2886_baseline	SSO	Amplitude	618.6585	343.9954	565.7817	430.1304
ZT12_G3_OA1	SSO	Frequency	407.3978	41.94953	397.7778	52.71467
ZT12_G3_OA1	SSO	Amplitude	386.1355	195.8682	390.6343	223.5977
ZT12_45_OA1	SSO	Frequency	442.7131	77.18562	428.8889	112.0187
ZT12_45_OA1	SSO	Amplitude	642.7155	388.6638	622.8234	465.6783
ZT12_2886_OA1	SSO	Frequency	349.041	14.16409	344.4444	16.47333
ZT12_2886_OA1	SSO	Amplitude	754.3368	352.145	728.0937	501.0738

Supplementary table 12: Two-sided wilcoxon signed-rank test on free fluctuation analysis in octopamine receptor mutant male mosquitoes. The Holms procedure was used to correct for multiple comparisons.

parameter	group1	group2	p	p.adj	p.format	p.signif	method
Frequency	G3|baseline	AGAP000045-|baseline	0.225387	0.23	0.22539	ns	Wilcoxon
Frequency	G3|baseline	AGAP002886-|baseline	3.65E-64	4.70E-63	< 2e-16	****	Wilcoxon
Frequency	G3|baseline	G3|OA1	7.19E-42	7.20E-41	< 2e-16	****	Wilcoxon
Frequency	G3|baseline	AGAP000045-|OA1	1.19E-45	1.30E-44	< 2e-16	****	Wilcoxon
Frequency	G3|baseline	AGAP002886-|OA1	1.77E-16	7.10E-16	< 2e-16	****	Wilcoxon
Frequency	AGAP000045-|baseline	AGAP002886-|baseline	3.91E-32	2.70E-31	< 2e-16	****	Wilcoxon
Frequency	AGAP000045-|baseline	G3|OA1	9.28E-34	7.40E-33	< 2e-16	****	Wilcoxon
Frequency	AGAP000045-|baseline	AGAP000045-|OA1	1.99E-40	1.80E-39	< 2e-16	****	Wilcoxon
Frequency	AGAP000045-|baseline	AGAP002886-|OA1	1.77E-17	8.80E-17	< 2e-16	****	Wilcoxon
Frequency	AGAP002886-|baseline	G3|OA1	1.91E-08	5.70E-08	1.90E-08	****	Wilcoxon
Frequency	AGAP002886-|baseline	AGAP000045-|OA1	1.29E-24	7.80E-24	< 2e-16	****	Wilcoxon
Frequency	AGAP002886-|baseline	AGAP002886-|OA1	2.44E-114	3.70E-113	< 2e-16	****	Wilcoxon

Frequency	G3|OA1	AGAP000045-|OA1	0.000768	0.0015	0.00077	***	Wilcoxon
Frequency	G3|OA1	AGAP002886-|OA1	7.01E-54	8.40E-53	< 2e-16	****	Wilcoxon
Frequency	AGAP000045-|OA1	AGAP002886-|OA1	1.88E-85	2.60E-84	< 2e-16	****	Wilcoxon
Amplitude	G3|baseline	AGAP000045-|baseline	1.94E-53	2.70E-52	< 2e-16	****	Wilcoxon
Amplitude	G3|baseline	AGAP002886-|baseline	2.58E-23	2.80E-22	< 2e-16	****	Wilcoxon
Amplitude	G3|baseline	G3|OA1	0.930438	1	0.93044	ns	Wilcoxon
Amplitude	G3|baseline	AGAP000045-|OA1	4.63E-19	4.60E-18	< 2e-16	****	Wilcoxon
Amplitude	G3|baseline	AGAP002886-|OA1	6.85E-55	1.00E-53	< 2e-16	****	Wilcoxon
Amplitude	AGAP000045-|baseline	AGAP002886-|baseline	3.31E-10	2.30E-09	3.30E-10	****	Wilcoxon
Amplitude	AGAP000045-|baseline	G3|OA1	7.70E-36	1.00E-34	< 2e-16	****	Wilcoxon
Amplitude	AGAP000045-|baseline	AGAP000045-|OA1	0.000627	0.0025	0.00063	***	Wilcoxon
Amplitude	AGAP000045-|baseline	AGAP002886-|OA1	0.883121	1	0.88312	ns	Wilcoxon
Amplitude	AGAP002886-|baseline	G3|OA1	1.51E-12	1.40E-11	1.50E-12	****	Wilcoxon
Amplitude	AGAP002886-|baseline	AGAP000045-|OA1	0.285161	0.86	0.28516	ns	Wilcoxon
Amplitude	AGAP002886-|baseline	AGAP002886-|OA1	3.41E-09	2.00E-08	3.40E-09	****	Wilcoxon
Amplitude	G3|OA1	AGAP000045-|OA1	1.44E-10	1.20E-09	1.40E-10	****	Wilcoxon
Amplitude	G3|OA1	AGAP002886-|OA1	3.04E-30	3.70E-29	< 2e-16	****	Wilcoxon
Amplitude	AGAP000045-|OA1	AGAP002886-|OA1	0.000448	0.0022	0.00045	***	Wilcoxon

Supplementary table 13: Summary of biophysical parameters extracted from frequency-modulated sweep responses in octopamine receptor mutant male mosquitoes upon exposure to octopamine

condition	State	parameters	mean	sd	median	mad
ZT12_G3_baseline	SSO	F0m	23.51784859	6.326110419	22.22667379	7.621279068
ZT12_G3_baseline	SSO	F0	358.995452	31.00066089	344.4432815	20.21407298
ZT12_G3_baseline	SSO	peak	374.8011826	37.59590604	356.361065	26.66236302
ZT12_G3_baseline	SSO	Q-factor	2.842306437	0.622395526	2.941332579	0.587244165
ZT12_G3_baseline	Quiesc	F0m	33.28834169	1.694667419	33.55677188	1.3813477
ZT12_G3_baseline	Quiesc	F0	380.9039464	3.764656137	380.8852662	2.54108984
ZT12_G3_baseline	Quiesc	peak	431.1391842	3.892978422	430.51736	3.873460983
ZT12_G3_baseline	Quiesc	Q-factor	1.51093507	0.040121883	1.495971287	0.028349027
ZT12_G3_OA1	SSO	F0m	-11.24262818	356.0904924	20.44884551	4.165593337
ZT12_G3_OA1	SSO	F0	402.8884611	139.1632794	382.3274199	46.89736817
ZT12_G3_OA1	SSO	peak	461.7241749	547.9159479	417.2433041	66.91408303
ZT12_G3_OA1	SSO	Q-factor	2.556991848	0.624290284	2.791319055	0.472191449
ZT12_G3_OA1	Quiesc	F0m	-16.25789799	354.9364274	14.31720488	18.76890668
ZT12_G3_OA1	Quiesc	F0	552.244119	676.2422417	544.5223919	108.0511881
ZT12_G3_OA1	Quiesc	peak	564.4976752	253.8425574	553.2625261	94.26433771
ZT12_G3_OA1	Quiesc	Q-factor	5.213208498	6.134360618	2.279820899	1.052797248
ZT12_45_baseline	SSO	F0m	25.55013792	6.400078903	22.42570389	1.782845503
ZT12_45_baseline	SSO	F0	368.1505237	13.77287596	369.0642466	16.92432678
ZT12_45_baseline	SSO	peak	380.1935982	14.3325964	379.9489354	14.27078122
ZT12_45_baseline	SSO	Q-factor	2.901545692	0.356815257	2.836535842	0.53937017
ZT12_45_baseline	Quiesc	F0m	37.63417542	0.523656508	37.60586443	0.519207167

ZT12_45_baseline	Quiesc	F0	419.649108	15.22065559	424.1329427	1.882931586
ZT12_45_baseline	Quiesc	peak	437.0842874	14.83811701	441.2462174	2.323145443
ZT12_45_baseline	Quiesc	Q-factor	2.529746694	0.07276627	2.545236467	0.023373275
ZT12_45_OA1	SSO	F0m	37.63534683	484.1195772	28.14991464	10.02336053
ZT12_45_OA1	SSO	F0	504.6169112	309.509325	450.2272485	87.54594651
ZT12_45_OA1	SSO	peak	526.7895975	298.6509025	469.8733279	90.00630002
ZT12_45_OA1	SSO	Q-factor	3.073653738	1.046768093	2.852257431	0.952760556
ZT12_45_OA1	Quiesc	F0m	198.9998939	2016.156695	1.544019455	52.65954394
ZT12_45_OA1	Quiesc	F0	691.3077382	577.3947373	402.9542416	170.1595252
ZT12_45_OA1	Quiesc	peak	1406.523034	1625.06253	588.3391798	331.3699991
ZT12_45_OA1	Quiesc	Q-factor	1.46226514	0.917101296	0.883436971	0.665583054
ZT12_86_baseline	SSO	F0m	27.84938439	6.784534765	25.84024427	2.108022987
ZT12_86_baseline	SSO	F0	393.6868964	21.31008696	397.5878599	15.12183381
ZT12_86_baseline	SSO	peak	424.0450198	49.94203717	409.8905449	32.67312733
ZT12_86_baseline	SSO	Q-factor	2.97326309	1.091230807	3.284563106	0.47774289
ZT12_86_OA1	SSO	F0m	29.66391556	5.408860141	30.01087734	4.702967292
ZT12_86_OA1	SSO	F0	380.2418808	30.29992285	382.8743902	24.87818308
ZT12_86_OA1	SSO	peak	415.7105045	53.98226036	393.8793946	58.18314299
ZT12_86_OA1	SSO	Q-factor	2.555127354	1.000974962	2.820140603	0.591384737

Supplementary table 14: Two-sided wilcoxon signed-rank test on frequency-modulated sweep analysis in octopamine receptor mutant males (pairwise comparisons). The Holms procedure was used to correct for multiple comparisons.

parameter	group1	group2	p	p.adj	p.format	p.signif	method
F0m	Baseline|wt	Baseline|45-	1.34E-05	4.00E-05	1.30E-05	****	Wilcoxon
F0m	Baseline|wt	Baseline|2886-	2.26E-16	2.90E-15	2.30E-16	****	Wilcoxon
F0m	Baseline|wt	1mM OA|wt	0.387475	0.77	0.39	ns	Wilcoxon

F0m	Baseline|wt	1mM OA|45-	7.52E-15	6.80E-14	7.50E-15	****		Wilcoxon
F0m	Baseline|wt	1mM OA|2886-	3.02E-27	4.20E-26	< 2e-16	****		Wilcoxon
F0m	Baseline|45-	Baseline|2886-	2.12E-08	8.50E-08	2.10E-08	****		Wilcoxon
F0m	Baseline|45-	1mM OA|wt	2.40E-09	1.40E-08	2.40E-09	****		Wilcoxon
F0m	Baseline|45-	1mM OA|45-	6.76E-09	3.40E-08	6.80E-09	****		Wilcoxon
F0m	Baseline|45-	1mM OA|2886-	7.58E-14	6.10E-13	7.60E-14	****		Wilcoxon
F0m	Baseline|2886-	1mM OA|wt	2.80E-16	3.40E-15	2.80E-16	****		Wilcoxon
F0m	Baseline|2886-	1mM OA|45-	6.20E-16	6.80E-15	6.20E-16	****		Wilcoxon
F0m	Baseline|2886-	1mM OA|2886-	3.18E-10	2.20E-09	3.20E-10	****		Wilcoxon
F0m	1mM OA|wt	1mM OA|45-	6.32E-16	6.80E-15	6.30E-16	****		Wilcoxon
F0m	1mM OA|wt	1mM OA|2886-	1.36E-33	2.00E-32	< 2e-16	****		Wilcoxon
F0m	1mM OA|45-	1mM OA|2886-	0.582645	0.77	0.58	ns		Wilcoxon
f0	Baseline|wt	Baseline|45-	2.30E-12	1.20E-11	2.30E-12	****		Wilcoxon
f0	Baseline|wt	Baseline|2886-	3.18E-41	3.80E-40	< 2e-16	****		Wilcoxon
f0	Baseline|wt	1mM OA|wt	1.26E-20	1.00E-19	< 2e-16	****		Wilcoxon
f0	Baseline|wt	1mM OA|45-	2.81E-62	3.90E-61	< 2e-16	****		Wilcoxon
f0	Baseline|wt	1mM OA|2886-	3.78E-19	2.60E-18	< 2e-16	****		Wilcoxon
f0	Baseline|45-	Baseline|2886-	1.16E-32	1.20E-31	< 2e-16	****		Wilcoxon
f0	Baseline|45-	1mM OA|wt	1.85E-06	5.60E-06	1.90E-06	****		Wilcoxon
f0	Baseline|45-	1mM OA|45-	1.42E-58	1.90E-57	< 2e-16	****		Wilcoxon
f0	Baseline|45-	1mM OA|2886-	4.34E-10	1.70E-09	4.30E-10	****		Wilcoxon
f0	Baseline|2886-	1mM OA|wt	0.054732	0.11	0.055	ns		Wilcoxon
f0	Baseline|2886-	1mM OA|45-	1.27E-40	1.40E-39	< 2e-16	****		Wilcoxon
f0	Baseline|2886-	1mM OA|2886-	1.52E-13	9.10E-13	1.50E-13	****		Wilcoxon
f0	1mM OA|wt	1mM OA|45-	1.23E-21	1.10E-20	< 2e-16	****		Wilcoxon
f0	1mM OA|wt	1mM OA|2886-	0.138722	0.14	0.139	ns		Wilcoxon

f0	1mM OA|45-	1mM OA|2886-	2.87E-63	4.30E-62	< 2e-16	****	Wilcoxon
peak	Baseline|wt	Baseline|45-	1.31E-05	5.20E-05	1.30E-05	****	Wilcoxon
peak	Baseline|wt	Baseline|2886-	3.29E-34	3.90E-33	< 2e-16	****	Wilcoxon
peak	Baseline|wt	1mM OA|wt	1.14E-16	9.10E-16	< 2e-16	****	Wilcoxon
peak	Baseline|wt	1mM OA|45-	3.95E-56	5.50E-55	< 2e-16	****	Wilcoxon
peak	Baseline|wt	1mM OA|2886-	7.49E-27	7.50E-26	< 2e-16	****	Wilcoxon
peak	Baseline|45-	Baseline|2886-	1.03E-26	9.30E-26	< 2e-16	****	Wilcoxon
peak	Baseline|45-	1mM OA|wt	1.53E-07	7.70E-07	1.50E-07	****	Wilcoxon
peak	Baseline|45-	1mM OA|45-	1.52E-58	2.30E-57	< 2e-16	****	Wilcoxon
peak	Baseline|45-	1mM OA|2886-	7.79E-11	4.70E-10	7.80E-11	****	Wilcoxon
peak	Baseline|2886-	1mM OA|wt	0.071545	0.21	0.072	ns	Wilcoxon
peak	Baseline|2886-	1mM OA|45-	3.31E-35	4.30E-34	< 2e-16	****	Wilcoxon
peak	Baseline|2886-	1mM OA|2886-	0.165819	0.33	0.166	ns	Wilcoxon
peak	1mM OA|wt	1mM OA|45-	1.72E-16	1.20E-15	< 2e-16	****	Wilcoxon
peak	1mM OA|wt	1mM OA|2886-	0.338248	0.34	0.338	ns	Wilcoxon
peak	1mM OA|45-	1mM OA|2886-	2.76E-29	3.00E-28	< 2e-16	****	Wilcoxon
Q	Baseline|wt	Baseline|45-	0.110713	0.44	0.1107	ns	Wilcoxon
Q	Baseline|wt	Baseline|2886-	5.71E-07	6.90E-06	5.70E-07	****	Wilcoxon
Q	Baseline|wt	1mM OA|wt	1.07E-05	0.00011	1.10E-05	****	Wilcoxon
Q	Baseline|wt	1mM OA|45-	0.038339	0.19	0.0383	*	Wilcoxon
Q	Baseline|wt	1mM OA|2886-	6.91E-09	9.00E-08	6.90E-09	****	Wilcoxon
Q	Baseline|45-	Baseline|2886-	8.28E-07	9.10E-06	8.30E-07	****	Wilcoxon
Q	Baseline|45-	1mM OA|wt	6.41E-05	0.00045	6.40E-05	****	Wilcoxon
Q	Baseline|45-	1mM OA|45-	0.417669	1	0.4177	ns	Wilcoxon
Q	Baseline|45-	1mM OA|2886-	0.001264	0.0076	0.0013	**	Wilcoxon
Q	Baseline|2886-	1mM OA|wt	3.53E-11	4.90E-10	3.50E-11	****	Wilcoxon
Q	Baseline|2886-	1mM OA|45-	0.874183	1	0.8742	ns	Wilcoxon
Q	Baseline|2886-	1mM OA|2886-	1.48E-11	2.20E-10	1.50E-11	****	Wilcoxon

Q	1mM OA|wt	1mM OA|45-	1.08E-05	0.00011	1.10E-05	****	Wilcoxon
Q	1mM OA|wt	1mM OA|2886-	0.509422	1	0.5094	ns	Wilcoxon
Q	1mM OA|45-	1mM OA|2886-	1.37E-05	0.00011	1.40E-05	****	Wilcoxon

Supplementary table 15: Summary of steady-state stiffness values extracted from force-step stimulation responses in octopamine receptor mutant male mosquitoes upon exposure to octopamine

condition	parameters	mean	sd	median	mad
ZT12_G3_ baseline	Steady-state stiffness	229.4069	140.9107	180.2018	36.50918
ZT12_45_ baseline	Steady-state stiffness	160.6101	24.17638	159.6484	34.58502
ZT12_2886_ baseline	Steady-state stiffness	156.1379	25.8627	150.6974	15.59826
ZT12_G3_OA1	Steady-state stiffness	488.5785	366.961	395.9188	280.4134
ZT12_45_OA1	Steady-state stiffness	302.5479	345.3858	177.5663	60.1543
ZT12_2886_OA1	Steady-state stiffness	145.6313	40.17464	136.9603	37.3214

Supplementary table 16: Two-sided wilcoxon signed-rank test on force-step stimulation analysis in octopamine receptor mutant males (pairwise comparisons). The Holms procedure was used to correct for multiple comparisons.

.y.	group1	group2	p	p.adj	p.format	p.signif	method
value	wt baseline	wt OA1mM	0.035556	0.39	0.0356	*	Wilcoxon
value	wt baseline	AGAP002886-baseline	0.020513	0.25	0.0205	*	Wilcoxon
value	wt baseline	AGAP002886-OA1mM	0.054079	0.54	0.0541	ns	Wilcoxon
value	wt baseline	AGAP000045-baseline	0.104895	0.73	0.1049	ns	Wilcoxon
value	wt baseline	AGAP000045-OA1mM	0.95913	1	0.9591	ns	Wilcoxon
value	wt OA1mM	AGAP002886-baseline	0.001243	0.017	0.0012	**	Wilcoxon
value	wt OA1mM	AGAP002886-OA1mM	0.002176	0.028	0.0022	**	Wilcoxon
value	wt OA1mM	AGAP000045-baseline	0.001088	0.016	0.0011	**	Wilcoxon
value	wt OA1mM	AGAP000045-OA1mM	0.064957	0.58	0.065	ns	Wilcoxon
value	AGAP002886-baseline	AGAP002886-OA1mM	0.382867	1	0.3829	ns	Wilcoxon
value	AGAP002886-baseline	AGAP000045-baseline	0.866511	1	0.8665	ns	Wilcoxon
value	AGAP002886-baseline	AGAP000045-OA1mM	0.120591	0.73	0.1206	ns	Wilcoxon
value	AGAP002886-OA1mM	AGAP000045-baseline	0.396892	1	0.3969	ns	Wilcoxon
value	AGAP002886-OA1mM	AGAP000045-OA1mM	0.072106	0.58	0.0721	ns	Wilcoxon

value	AGAP000045-baseline	AGAP000045-OA1mM	0.234499	1	0.2345	ns	Wilcoxon
