# Supplementary material for: Hearing of malaria mosquitoes is modulated by a beta-adrenergic-like octopamine receptor which serves as insecticide target
Source: Nat Commun. 2023 Jul 19;14:4338. doi: 10.1038/s41467-023-40029-y (PMC10356864; doi:10.1038/s41467-023-40029-y)
Supplement: Supplementary file 3 — Reporting Summary [file 41467_2023_40029_MOESM3_ESM.pdf]

nature portfolio

Corresponding author(s): Marta Andres

Last updated by author(s): May 4, 2023

Reporting Summary

Nature Portfolio wishes to improve the reproducibility of the work that we publish. This form provides structure for consistency and transparency in reporting. For further information on Nature Portfolio policies, see our Editorial Policies and the Editorial Policy Checklist.

Statistics

For all statistical analyses, confirm that the following items are present in the figure legend, table legend, main text, or Methods section.

n/a	Confirmed	
☐	☒	The exact sample size (n) for each experimental group/condition, given as a discrete number and unit of measurement
☐	☒	A statement on whether measurements were taken from distinct samples or whether the same sample was measured repeatedly
☐	☒	The statistical test(s) used AND whether they are one- or two-sided<br>*Only common tests should be described solely by name; describe more complex techniques in the Methods section.*
☒	☐	A description of all covariates tested
☐	☒	A description of any assumptions or corrections, such as tests of normality and adjustment for multiple comparisons
☐	☒	A full description of the statistical parameters including central tendency (e.g. means) or other basic estimates (e.g. regression coefficient) AND variation (e.g. standard deviation) or associated estimates of uncertainty (e.g. confidence intervals)
☐	☒	For null hypothesis testing, the test statistic (e.g. F, t, r) with confidence intervals, effect sizes, degrees of freedom and P value noted<br>*Give P values as exact values whenever suitable.*
☒	☐	For Bayesian analysis, information on the choice of priors and Markov chain Monte Carlo settings
☒	☐	For hierarchical and complex designs, identification of the appropriate level for tests and full reporting of outcomes
☒	☐	Estimates of effect sizes (e.g. Cohen's d, Pearson's r), indicating how they were calculated

Our web collection on statistics for biologists contains articles on many of the points above.

Software and code

Policy information about availability of computer code

Data collection	Spike 2 version 10
Data analysis	R 4.2.2 (packages DESeq2 1.36.0, MetaCycle 1.2.0), Python 3.8 (packages: NEAT 3.0, goatools 0.4.7), Mathematica v.13.1, FastQC 0.11.9, MultiQC 1.10, Kallisto 0.46.0

For manuscripts utilizing custom algorithms or software that are central to the research but not yet described in published literature, software must be made available to editors and reviewers. We strongly encourage code deposition in a community repository (e.g. GitHub). See the Nature Portfolio guidelines for submitting code & software for further information.

Data

Policy information about availability of data

All manuscripts must include a data availability statement. This statement should provide the following information, where applicable:

- Accession codes, unique identifiers, or web links for publicly available datasets
- A description of any restrictions on data availability
- For clinical datasets or third party data, please ensure that the statement adheres to our policy

The RNA-Seq raw data generated in this study have been deposited in the YY database under accession code ZZ [add hyperlink here]. The processed RNA-Seq data and raw data underlying graphs are provided in the Supplementary Information/Source Data file. All data used for analyses in this paper, as well as further details regarding experimental or analytical procedures, are available from the authors.

Research involving human participants, their data, or biological material

Policy information about studies with human participants or human data. See also policy information about sex, gender (identity/presentation), and sexual orientation and race, ethnicity and racism.

Reporting on sex and gender	N/A
Reporting on race, ethnicity, or other socially relevant groupings	N/A
Population characteristics	N/A
Recruitment	N/A
Ethics oversight	N/A

Note that full information on the approval of the study protocol must also be provided in the manuscript.

Field-specific reporting

Please select the one below that is the best fit for your research. If you are not sure, read the appropriate sections before making your selection.

☒ Life sciences ☐ Behavioural & social sciences ☐ Ecological, evolutionary & environmental sciences

For a reference copy of the document with all sections, see nature.com/documents/nr-reporting-summary-flat.pdf

Life sciences study design

All studies must disclose on these points even when the disclosure is negative.

Sample size	Samples sizes for LDV experiments were determined based on published data on Dipteran antennal LDV measurements:<br>Su, M. P., Andrés, M., Boyd-Gibbins, N., Somers, J. & Albert, J. T. Sex and species specific hearing mechanisms in mosquito flagellar ears. Nature Communications 9, 3911 (2018).<br>Göpfert, M. C., Humphris, A. D. L., Albert, J. T., Robert, D. & Hendrich, O. Power gain exhibited by motile mechanosensory neurons in Drosophila ears. Proc Natl Acad Sci U S A 102, 325–330 (2005).<br>Effertz, T., Nadrowski, B., Piepenbrock, D., Albert, J. T. & Gopfert, M. C. Direct gating and mechanical integrity of Drosophila auditory transducers require TRPN1. Nat Neurosci 15, 1198–1200 (2012).<br>Three replicates for RNA-Seq experiments is considered as the standard.
Data exclusions	One of the replicates of the RNA-Seq collected for ZT12 was deemed unsuitable for further analyses and was removed from the dataset. The expression levels of mechanosensory genes known to be highly expressed in the Johnston's organ of insects were extremely low, and therefore we decided to exclude this replicate due to its low quality. Apart from that, no data were excluded from the analysis.
Replication	All attempts at replication were successful. The effects of octopamine in the fibrillae erection has been tested by 4 independent people in the lab. The experiments to analyse the effects of octopamine on mosquito audition were collected along two years, including many different generations of mosquitoes, and the results were always replicated.
Randomization	Not relevant because of the nature of the study. We could not inject compounds in a random order because the order of injection matters. For that, we always compare the effects of injected compounds and injected controls.
Blinding	The analysis of the auditory test results (free-fluctuations, force-step responses, frequency-modulated sweep responses) both for wildtype and mutant mosquitoes, were analysed computationally and the results of the analysis have been plotted in the manuscript, so the data have been “blinded” computationally. No manual curation was performed. For the analysis of the fibrillae erection in mutant animals, the observer was blinded regarding the phenotype of the animals. Numbers were allocated to the glass vials where mosquitoes were kept by a different person. After the fibrillae erection state was assessed, phenotypes were allocated to the glass vials.

Reporting for specific materials, systems and methods

We require information from authors about some types of materials, experimental systems and methods used in many studies. Here, indicate whether each material, system or method listed is relevant to your study. If you are not sure if a list item applies to your research, read the appropriate section before selecting a response.

Materials & experimental systems

n/a	Involved in the study	
☒	☐	Antibodies
☒	☐	Eukaryotic cell lines
☒	☐	Palaeontology and archaeology
☐	☒	Animals and other organisms
☒	☐	Clinical data
☒	☐	Dual use research of concern
☒	☐	Plants

Methods

n/a	Involved in the study	
☒	☐	ChIP-seq
☒	☐	Flow cytometry
☒	☐	MRI-based neuroimaging

Animals and other research organisms

Policy information about studies involving animals; ARRIVE guidelines recommended for reporting animal research, and Sex and Gender in Research

Laboratory animals	Anopheles gambiae mosquitoes of Kisumu and G3 strains were used in the study, 3-5 days old.
Wild animals	No wild animals were used in this study.
Reporting on sex	Mosquitoes were assigned to being males or females at pupae stages based on their terminal segments, which show clear sexual dimorphisms. RNA-Seq analysis was performed independently in males and females, and findings are reported separately for both sexes. Auditory tests were performed both in males and females and results are reported in the manuscript separately.
Field-collected samples	No field collected samples were used in this study.
Ethics oversight	No ethical approval was required.

Note that full information on the approval of the study protocol must also be provided in the manuscript.
