# Supplementary material for: Hearing of malaria mosquitoes is modulated by a beta-adrenergic-like octopamine receptor which serves as insecticide target
Source: Nat Commun. 2023 Jul 19;14:4338. doi: 10.1038/s41467-023-40029-y (PMC10356864; doi:10.1038/s41467-023-40029-y)
Supplement: Supplementary file 4 — Description of Additional Supplementary Files [file 41467_2023_40029_MOESM4_ESM.pdf]

Description of Additional Supplementary Files

Supplementary Data 1
Description: Kallisto estimation of counts for male and female JO samples. This spreadsheet contains the output of Kallisto, that is, the raw (prior to any analyses) expression count estimates for each transcript for each sample collected from male and female JOs. Note that these counts are unprocessed and nonnormalised and thus differ from the expression counts of the respective transcripts included in previous tables. As before, the id column holds the VectorBase identities for each transcript. Columns M_ZT0, M_ZT0.1,…, M_ZT20.2 contain the male expression counts and similarly columns F_ZT0, F_ZT0.1, …, F_ZT20.2 contain the female expression counts.

Supplementary Data 2
Description: Genes expressed in the male Johnston's Organ (JO). This spreadsheet contains all the transcripts that were found to be expressed in the male JO. The first column holds the VectorBase identities of the transcripts and the remaining columns hold the expression counts at each of six circadian time points (ZT0, ZT4, ZT8, ZT12, ZT16, and ZT20). Each circadian time point was sampled three times (e.g. ZT0, ZT0.1, ZT0.2). One of the replicates collected for ZT12 was deemed unsuitable for further analyses and was removed from the dataset. Expression of each gene was assessed statistically as follows: A Poisson distribution for the noise counts of transcripts was defined (with average noise counts = 11.8) and the mean expression counts of each transcript was compared against it, filtering out transcripts whose expression counts could have arisen solely by noise. For more information on the procedure see the Methods section.

Supplementary Data 3
Description: Genes expressed in the female JO. As in Supplementary table 1, this spreadsheet contains all the transcripts that were found to be expressed in the female JO.

Supplementary Data 4
Description: Neuromodulatory genes expressed in the male JO. This spreadsheet contains the subset of the male transcripts included in Supplementary table 1 that were assigned with specific gene ontologies (GOs; categorisations of gene products that link gene products to one or more functions) of interest to this study. For more information on the GOs used here, as well as the GO-mining procedure, see the Methods section. As in Supplementary table 1, the first column holds the VectorBase identities of the transcripts. The column titled gene holds the VectorBase identity of the gene producing the specific transcript. The column gene_symbol assigns a gene symbol to the transcript, if one exists, otherwise it assigns the VectorBase identity of the gene as a symbol for the transcript. GO holds the gene ontological class of each transcript. The columns ZT0 – ZT20.2 contain the expression counts of transcripts (as in Supplementary table 1); ZT_avg holds the transcripts' mean expression (mean(ZT0, ZT0.1,…,ZT20.2)), and log_ZT_avg holds the log10 of the mean expression (that is, log10(ZT_avg)).

Supplementary Data 5
Description: Neuromodulatory genes expressed in the female JO. Similar to Supplementary table 3, this spreadsheet contains the subset of the female transcripts included in Supplementary table 2 that were assigned with specific GOs.

Supplementary Data 6
Description: Differential expression analysis of males compared to females. This spreadsheet contains the results of the differential expression analysis, conducted with DEseq2, comparing male and female JO transcript expression (see Methods section for more information). As above, id holds the VectorBase identity of the transcript. The column baseMean calculates the mean expression of a

transcript's counts, taken across all samples (both male and female). The column log2FoldChange estimates the effect size; it calculates, for each transcript, the logarithm (base 2) of the ratio ('FoldChange') of male expression counts to female expression counts. For example, a transcript that is expressed twice as much in the male as in the female would yield a log2FoldChange of 1, whereas a transcript that is expressed half as much as in the male as it is in the female would yield a log2FoldChange of -1. The column lfcSE estimates the standard error for the log2 fold change. The column stat contains the test statistic for each transcript (log2 fold change divided by its standard error), that is compared to a standard normal distribution. The statistical test employed by DEseq2 for this comparison is called the Wald test, and it is a two-tailed test. Finally, the columns pvalue and padj represent the p-value and p-value that is adjusted for multiple comparisons (via the Benjamini-Hochberg method), for each transcript.

Supplementary Data 7
Description: Transcripts exhibiting cyclical expression in the male JO. This spreadsheet contains the results of the cycling analysis conducted with JTK cycle on male JO samples. As above, the column id holds the VectorBase identities for each transcript. PER, LAG, and AMP estimate, for the cycling expression of each transcript, the period, phase, and amplitude of oscillation respectively. The period of the expression's oscillation is the time it takes for one oscillation to complete. Phase is the time point at which the transcript's expression reaches its maximum value. The amplitude of the oscillation is the difference between the transcript's maximum expression and baseline expression values. The columns Pvalue and BH.Q hold the p-value, for the statistical test of a transcript exhibiting cyclical expression, and the adjusted for multiple comparisons p-value (via the Benjamini-Hochberg method), respectively. The statistical test employed by JTK cycle for the identification of cycling transcripts is a combination of the Jonckheere–Terpstra test and Kendall's τ test; it is a two-tailed test.

Supplementary Data 8
Description: Transcripts exhibiting cyclical expression in the female JO. As in Supplementary table 6, this spreadsheet contains the results of the cycling analysis conducted on the female JO samples.
